# Mechanotransduction-Mediated Expansion of Rabbit Vocal Fold Epithelial Cells via ROCK Inhibition and Stromal Cell-Derived Paracrine Signals

**DOI:** 10.3390/cells14181412

**Published:** 2025-09-09

**Authors:** Samjhana Thapa, Joo Hyun Kim, Jun Yeong Jeong, Sung Sik Hur, Seung Won Lee, Yongsung Hwang

**Affiliations:** 1 Soonchunhyang Institute of Medi-Bio Science (SIMS), Soonchunhyang University, Cheonan-si 31151, Republic of Korea; samjhanathp@gmail.com (S.T.); noirsky@naver.com (J.H.K.); sstahur@gmail.com (S.S.H.); 2 Department of Integrated Biomedical Science, Soonchunhyang University, Asan-si 31538, Republic of Korea; 3 Department of Otorhinolaryngology-Head and Neck Surgery, College of Medicine, Soonchunhyang University, Cheonan Hospital, Cheonan-si 31151, Republic of Korea; 4 Department of Otorhinolaryngology-Head and Neck Surgery, College of Medicine, Soonchunhyang University, Bucheon Hospital, Bucheon-si 14584, Republic of Korea; 136076@schmc.ac.kr (J.Y.J.)

**Keywords:** self-renewal, Rho kinase inhibitor (ROCKi), cytoskeletal remodeling, focal adhesion, mechanotransduction

## Abstract

Therapeutic advances for vocal fold (VF) disorders are limited by the scarcity of VF-derived epithelial cells (VFEs). Despite their substantial self-renewal capability in vivo, VFEs expand for only a few passages in vitro before succumbing to growth arrest. This has led to the extensive use of alternative cellular sources that are not exposed to physiological stresses of phonation. To address this, we developed an ideal culture strategy that enables long-term expansion of rabbit VFEs (rbVFEs), by utilizing Rho kinase inhibitor (ROCKi), epidermal growth factor (EGF), and mitomycin-treated STO cells or its conditioned media (STO-CM). ROCKi only could support short-term proliferation, and rbVFEs eventually underwent senescence. Further enhancement to ROCKi-containing media with EGF or STO-CM promoted sustained proliferation of rbVFEs. Mechanistically, non-self-renewing rbVFEs exhibited cytoskeletal remodeling associated with increased nuclear YAP localization, elevated focal adhesion, and higher traction forces, whereas self-renewing rbVFEs had cytoplasmic YAP retention, decreased adhesion, and reduced cellular tension. Our optimized culture strategy provides a robust supply of rbVFEs for advancing regenerative approaches in VF research.

## 1. Introduction

The vocal fold (VF) consists of an outermost epithelium composed of 5–7 layers of stratified squamous epithelial cells [1]. Although the epithelium plays an important role in tissue homeostasis and disease progression, very few studies have focused on the role of VF epithelium owing to its poor self-renewal ability [2]. Several researchers have isolated and expanded VFEs from porcine [2,3], rabbit [4] and human [5] VF specimens; however, they could not maintain their proliferative ability beyond passage 4. Although efforts have been made to immortalize human VFEs [6] and differentiate induced pluripotent stem cells (iPSCs) into VFEs [1], the originality of the cell and its functional relevance remain a significant concern [5]. Given the unique biomechanical properties of the VF, which endures continuous tensile, shear, and impact stress during phonation [7], primary VF epithelial cells are the most suitable cell type for VF studies [8], especially mechanobiological studies [9].

Rho kinase (ROCK) proteins are immediate downstream effectors of Rho-mediated signaling pathways that regulate various cellular activities, such as cytoskeletal reorganization, gene expression, and cell cycle progression [10]. Thus, they serve as potent inhibitors of differentiation and apoptosis [11], and as promoters of cell proliferation [12]. Additionally, ROCKi induce progenitor clone formation by modulating key genes involved in basal cytoskeleton formation, cell–cell adhesion, and cell–extracellular matrix (ECM) interactions [13]. Given these properties, ROCKi represent a promising strategy for expanding primary VFEs while maintaining their undifferentiated state. However, ROCKi cause the loss of ROCK1 and ROCK2, thus hampering actomyosin contractility, reducing stress fibers, and leading to a flattened cell shape and enlarged morphology that is typical of cellular senescence [14]. Senescence gradually leads to an increase in cell size [15] and cell flattening [16], which in turn impairs cell function, proliferative ability, and protein synthesis [17]. Cellular senescence has also been linked to increased traction stress in endothelial cells [18]. Several studies have reported that senescent cells express stressed f-actin and mature focal adhesions as adaptations to the non-proliferative state [19]. Multiple cellular behaviors, including morphological changes and focal adhesion formation, are linked to cell-generated traction forces [20]. Senescent EC exhibit increased traction forces and thicker actin bundles [18].

Furthermore, ROCKi in combination with growth-arrested fibroblast feeder layers have been widely used to isolate and expand both normal and cancerous epithelial cells from various tissues [21,22]. Epithelial cells cultured under these conditions exhibit a stem-like state, which enables long-term proliferation [21]. Feeder cells produce growth factors, adhesion molecules, and extracellular matrices [23]. Some of the most abundant cytokine proteins expressed by feeder (STO) cells are activin A, hepatocyte growth factor (HGF), insulin-like growth factors (IGF-1 and IGF-2), insulin-like growth factor-binding protein (IGFBP)-6, macrophage colony-stimulating factor (CSF-1), and pigment epithelium-derived factor (PEDF) [24]. Soluble factors from fibroblast feeders contribute to niche formation and support the self-renewal or proliferation of the surrounding cells [25] and help to maintain epithelial cells in an undifferentiated state [26]. Additionally, feeder cells promote epithelial colony formation [27] by preventing fibroblast invasion and outgrowth [28]. The effectiveness of STO feeders in supporting epithelial cell expansion from multiple species makes them promising candidates for VFEs cultures.

EGF is a key growth factor that plays a vital role in regulating the proliferation and differentiation of stem cells [29]. Although certain stem cells differentiate in response to EGF, Tropepe et al. demonstrated that EGF supports the self-renewal of murine neural stem cells (NSCs) [30]. Leydon et al. demonstrated that the EGF pathway is activated during the active re-epithelialization phase of VF wound healing [31]. Based on the findings of Leydon et al. [31] and extensive research highlighting the mitogenic effects of EGF, we hypothesized that incorporating exogenous EGF into an in vitro culture system could enhance the rapid proliferation of VF epithelial cells. This study aimed to develop a novel cell culture strategy to support the rapid proliferation and self-renewal of rabbit VF tissue-derived epithelial cells (rbVFEs). We further investigated the effects of ROCKi, STO-conditioned medium (STO-CM), and EGF on enhancing rbVFE proliferation. Additionally, we explored how these factors influence cytoskeletal remodeling and cellular forces during self-renewal.

## 2. Materials and Methods

### 2.1. Animal Preparation and VF Tissue Harvesting

Male New Zealand White breeder rabbits (Samtako Bio, Osan, Gyeonggi-do, Republic of Korea) weighing 2.5–3 kg and aged 14 weeks were used in this study. The animals were housed at the Soonchunhyang University Animal Center. Each rabbit was euthanized using CO_2_, and the larynx was immediately harvested and immersed in sterile 1× phosphate-buffered saline (PBS [10×], pH 7.4; Cat. # 70011044; Gibco™, Thermo Fisher Scientific, Waltham, MA, USA) diluted in distilled water. The paralaryngeal fat and connective tissue were removed, and an incision was made between the arytenoid cartilages to expose the VFs. The outermost mucosal layer was then carefully excised using a scalpel. All procedures followed the ethical guidelines approved by the Institutional Animal Care and Use Committee of Soonchunhyang University Bucheon Hospital (SCHBCA2022-09-1).

### 2.2. Histological Staining of Vocal Fold Tissue

The harvested larynges were immediately immersed in 4% paraformaldehyde (CellNest 4% Paraformaldehyde in PBS, Cat. # CNP015-0500, Cheonan, Chungnam, Republic of Korea) and stored at 4 °C. Prior to histological processing, the fixed laryngeal specimens were washed with 1× PBS and dehydrated using a graded ethanol series (60%, 70%, 80%, 95%, 100%, and 100%) (Daejung Ethyl Alcohol 95%, Denatured, Cat. # 4119-4410, Daejung Chemicals & Metals Co., Ltd., Siheung-si, Gyeonggi-do, Republic of Korea) followed by treatment with xylene (S3-Histo™ Xylene Substitute, Cat. # 4322, BBC Biochemical, Mount Vernon, WA, USA). The dehydrated larynges were then embedded in paraffin and sectioned into 5 µm thick slices using a microtome. The paraffin sections were stained with hematoxylin (Mayer’s Modified Hematoxylin Solution, Cat. # ab220365, Abcam, Cambridge, MA, USA) and eosin (Eosin Y Alcoholic, Cat. # MA0101015, BBC Biochemical, Mount Vernon, WA, USA) for routine histological examination. Additionally, Masson’s Trichrome staining was performed using the Masson’s Trichrome Stain Kit (Cat. # BAQ086, G Biosciences, St. Louis, MO, USA) to assess collagen distribution.

### 2.3. Media Composition for Culturing VF Epithelial Cells

The culture media consisted of Dulbecco’s Modified Eagle’s Medium (Corning^®^ 500 mL DMEM, Cat. # 10-013-CV, Corning Life Sciences, Corning, NY, USA) supplemented with 10% FBS (Corning^®^ Fetal Bovine Serum, 500 mL, Premium, United States Origin, Cat. # 35-015-CV, Corning Life Sciences, Corning, NY, USA), 1% Penicillin-Streptomycin (Gibco™ Penicillin-Streptomycin, 10,000 U/mL, Cat. # 15140122, Life Technologies, Grand Island, NY, USA), and Glutamax I (Gibco™ GlutaMAX™ Supplement, Cat. # 35050-061, Life Technologies Corporation, Grand Island, NY, USA). This medium was further mixed with Ham’s F12 nutrient mix (Gibco™ Ham’s F-12 Nutrient Mix, Cat. # 11765054, Grand Island, NY, USA) in a 3:1 ratio and supplemented with a final concentration of 5 μg/mL insulin (Insulin from bovine pancreas, Cat. # 15500, Sigma-Aldrich, St. Louis, MO, USA), 250 ng/mL Amphotericin B (Life Technologies, Cat. # 152900263, Burlington, ON, Canada), 10 μg/mL Gentamicin (Gentamicin Reagent Solution, Cat. # 15750060, Life Technologies, Burlington, ON, Canada), and 25 ng/mL hydrocortisone (Hydrocortisone BioReagent, suitable for cell culture, Cat. # H0888, Sigma-Aldrich, St. Louis, MO, USA). The medium was sterile filtered, stored at 4 °C, and then used within 2 months of preparation. Finally, 5 ng/mL EGF (Gibco™ Human EGF Recombinant Protein, Cat. # PHG0313, Gibco, Grand Island, NY, USA) and 10 μM ROCKi (Y27632 Dihydrochloride, Cat. # 1293823, PeproTech, Rocky Hill, NJ, USA) were added to the medium according to the experimental setup.

### 2.4. Mitomycin C (MMC) Treatment to STO Cells

Sandos inbred mouse (SIM) 6-thioguanine-resistant, ouabain-resistant (STO) cell lines were purchased from ATCC (Cat. # CRL-1503, Manassas, VA, USA) and plated at a seeding density of 10 K/cm^2^ in 0.1% gelatin-coated (Gelatin from porcine skin, Cat. # G6144, Sigma-Aldrich, St. Louis, MO, USA) tissue culture plates (TCP). Mitomycin C obtained from *Streptomyces caespitosus* (Cat. # M0503 Sigma-Aldrich, St. Louis, MO, USA) was diluted to a final concentration of 10 µg/mL in DMEM supplemented with 10% FBS and 1% P/S. Once STO cells reached confluence, they were incubated with MMC containing media for 4 h in a 37 °C incubator with 5% CO_2_. Cells were washed at least six times with 1× PBS and trypsinized with 0.05% trypsin-EDTA (Gibco™ Trypsin-EDTA [0.05%], phenol red, Cat. # 25200062, Life Technologies, Burlington, ON, Canada). The MMC-treated STO cells were stored in an LN2 tank until use. To culture rbVFEs, growth-arrested STO cells were plated at a seeding density of 35 K/cm^2^ in 0.1% gelatin-coated TCP and incubated for at least 24 h before the initial seeding of rbVFEs.

### 2.5. Preparation of MMC Treated STO-Conditioned Media

MMC-treated STO cells were plated at a seeding density of 70 K/cm^2^ in a 90 cm^2^ TCP, and 10 mL of DMEM supplemented with 10% FBS and 1% P/S was added. Conditioned media were collected after 72 and 144 h, mixed, centrifuged at 300× *g* for 5 min, and then sterile filtered using a 0.22 µm vacuum filtration system (Corning^®^ 500 mL Vacuum Filter Storage Bottle System, 0.22 µm Pore 33.2 cm^2^ CA Membrane, Cat. # 430769, Corning, NY, USA). The media were further supplemented with 1% Glutamax I, and this formulation was mixed with Ham’s F12 Nutrient Mix in a 3:1 ratio and supplemented to a final concentration of 5 mg/mL insulin, 250 ng/mL amphotericin B, 10 μg/mL gentamicin, and 25 ng/mL hydrocortisone. The conditioned medium containing these growth factors was aliquoted into 15 or 50 mL conical tubes and stored at −80 °C. When needed, the medium was thawed and mixed with a freshly prepared medium (as described in Section 2.3) in a 3:1 ratio. Finally, 5 ng/mL EGF or 10 μM ROCKi was added according to the experimental setup. The resulting conditioned media were stored at 4 °C for up to 2 weeks.

### 2.6. VF Cell Isolation and Culture

The mucosal layer was cut from the VFs, rinsed with 100% ethanol for 3–5 s and rinsed twice with PBS. The tissues were finely minced with a scalpel and placed with 4 mL of digestion medium in a 15 mL conical tube. The digestion medium consisted of collagenase/hyaluronidase (C/H); Cat. # 07912, StemCell Technologies, Vancouver, BC, Canada and Dispase (Corning^®^ Dispase, Cat. # 354235, Sigma-Aldrich, St. Louis, MO, USA). Briefly, C/H was diluted in media (prepared as described earlier in Section 2.3) at a 1:9 ratio. Then, three parts of this formulation were mixed with 1 mL of dispase, leading to a final ratio of 0.3:2.7:1 (C/H: media: dispase). The tube containing minced VF tissue in digestion media was placed on a rocking platform for 3 h in a 37 °C incubator with 5% CO_2_. The digested tissue was centrifuged at 500× *g* for 5 min, resuspended in DMEM (10% FBS, 1% p/s and 1% glutamax) and filtered through 100-µm cell strainer and pelleted again by centrifuging at 300× *g* for 5 min. The cells were seeded on top of an MMC-treated STO feeder layer. After cell isolation and seeding, rbVFE colonies were visible within 24 h and reached confluence within a week of culture.

### 2.7. Differential Adhesion-Based Removal of Fibroblasts from the Co-Culture

To remove the STO cells and overgrown fibroblasts from the co-culture, we followed the protocols described by Mizuta et al. [4] and Ling et al. [5] based on the differential adhesion properties of epithelial cells and fibroblasts. Cell culture plates containing a mixture of STO cells, VF epithelial cells, and fibroblasts were incubated with 0.05% trypsin-EDTA for 1–2 min at room temperature. Epithelial cells adhered tightly to the TCP compared to fibroblasts; thus, within 1–3 min, fibroblasts started to detach, while epithelial cells remained adherent (Figure S2A). STO cell and VF fibroblast detachments were constantly observed under a phase-contrast microscope. When the fibroblasts around the epithelial cell colonies started rounding up (usually within 1–3 min), the plate was gently tilted, and trypsin was suctioned out. The plates were washed twice with PBS to remove detached cells. The cells remaining on the TCP were further incubated with trypsin-EDTA for 7–10 min in a 37 °C incubator, collected, and further processed for MACS.

### 2.8. Magnetic-Activated Cell Sorting of VF-Derived Cells

Indirect MACS sorting was performed using a CD44 antibody (CD44 Monoclonal Antibody [Hermes-1], Rat monoclonal, Cat. # MA4400, ThermoFisher Scientific, Waltham, MA, USA, at dilution 1:50). MACS buffer was prepared with 0.5% BSA and 2 mM EDTA (EDTA, Disodium Dihydrate, Biotechnology Grade, Cat. # EDT001, Bioshop, Burlington, ON, Canada) diluted in PBS. Briefly, 2 × 10^6^ cells were suspended in 100 µL MACS buffer containing CD44 antibody at 1:50 dilution, and the cells were incubated for 30 min at 4 °C. The cells were then washed with MACS buffer and incubated with goat anti-rat IgG microbeads (Cat. # 130-048-501, Miltenyi Biotec, Bergisch Gladbach, Germany) at 1:5 dilution (20 µL microbeads in 80 µL MACS buffer) for 15 min at 4 °C. The cells were washed and passed through an MS column (Cat. # 130-042-201, Miltenyi Biotec, Bergisch Gladbach, Germany) to retain CD44-labeled cells. CD44-positive cells were considered fibroblasts, whereas CD44-negative populations were considered epithelial cells. Once purified, rbVFEs were passaged on day 4 post-seeding at a density of 10 K cells/cm^2^. For experimental purposes, cells at passages 4–7 were used.

### 2.9. Calculation of Population Doubling (PD) and PD Time

rbVFEs were cultured in different culture conditions at a seeding density of 10 K cells/cm^2^. On day 3, the cells were trypsinized and counted using a hemacytometer. The initial number of cells used for seeding was recorded during cell seeding. Population doubling (PD) was calculated using the formula PD = [log (N_f_) − log (N_i_)]/log2, where N_f_ is the final number of cells harvested, and N_i_ is initial number of cells seeded.

Population doubling time (PDT) was calculated using the formula PDT = (t)/PD, where (t) is the culture duration (in days or hours), and PD is population doubling.

Cumulative population doubling (CPD) was calculated as the sum of all individual PDs: Cumulative PD = ∑PD (addition of all PD).

### 2.10. Immunofluorescence Staining

Cells were fixed with 4% PFA for 15 min at room temperature (RT), washed with PBS, and permeabilized with 0.2% Triton-X 100 (TRITON ^®^X-100, Biotechnology Grade, Cat. # TRX777, Bioshop, Burlington, ON, Canada) diluted in PBS for 10 min at RT. The samples were washed with PBS and blocked with blocking buffer for 30 min at RT. The blocking buffer consisted of 3% bovine serum albumin BSA (ALBUMIN, BOVINE SERUM, Cat. # ALB001, Bioshop, Burlington, ON, Canada) and 0.05% TWEEN^®^ 20 (Cat. # P1379-100ML Sigma-Aldrich, St. Louis, MO, USA) in PBS. Primary antibodies (primary antibodies information provided in Supplementary Table S2) were diluted in blocking buffer and incubated overnight at 4 °C. The cells were washed, incubated with secondary antibodies diluted in blocking buffer (secondary antibodies information is provided in Supplementary Table S3), and incubated for 1 h at RT. Images were captured using EVOS™ M7000 Imaging System, Cat. # AMF7000, Invitrogen, Thermo Fisher Scientific, Waltham, MA, USA. Negative control specimens were processed similarly to the stained specimens, except for incubation with primary antibodies.

### 2.11. Cell Proliferation Assay

The rbVFEs were seeded at 10 K/cm^2^ in 0.1% gelatin-coated 96-well plates for five different experimental groups. After 10 h of cell attachment, the medium was suctioned, the CellVia Enhanced Cell Viability Assay Kit (Cat. # LF-EZ1001A Abfrontier, Seoul, Republic of Korea) was diluted in a 1:10 ratio with media, and 100 μL of the mixture was added to each well; the cells were incubated for 2 h in a 37 °C incubator with 5% CO_2_. After incubation, the plates were gently shaken to homogenize the medium color, and the absorbance was measured at 450 nm using a microplate reader (Agilent BioTek Epoch Microplate Spectrophotometer, Agilent Technologies, Santa Clara, CA, USA). The medium-containing cell via reagent was suctioned out; fresh media was added to each well, and the plate was returned to the 37 °C incubator with 5% CO_2_. Absorbance was measured at 24, 72, and 120 h using the same plate, and the absorbance values at different time points were normalized to the absorbance value at 10 h, which was considered day 0.

### 2.12. EdU Assay

The rbVFEs were seeded under the respective culture conditions at a density of 10 K/cm^2^. After 36 h, the cells were incubated with EdU components using the EdU Alexa Fluor 488 Imaging Kit (Cat. # C10337, Invitrogen, Thermo Fisher Scientific, Waltham, MA, USA). Briefly, 10 mM EdU was dissolved in respective media to make 20 µM of EdU solution. From each well, half of the media (200 µL in each well of 48 well plate) and 200 µL of the 20 µM EdU solution were added in each well. The cells were incubated for 2 h in a 37 °C incubator. Cells were fixed with 4% PFA for 15 min at RT. The cells were permeabilized with 0.5% Triton X-100 in 3% BSA for 20 min at RT. The EdU detection cocktail consisted of 1X Click-iT™ reaction buffer, CuSO_4_, Alexa Fluor™ azide, and reaction buffer additive, prepared according to the manufacturer’s instructions. The total DNA was stained with Hoechst 33,342 (Cat. # H3570, Thermo Fisher Scientific) at a dilution of 1:2000 in 1X PBS. The number of EdU- and Hoechst-positive cells were counted using the ImageJ software (version 1.54f). Images were captured using the EVOS M7000 Imaging System. The percentage of cells in the S-phase was calculated using the formula %EdU-positive cells = (Number of EdU-positive cells/total Hoechst-positive cells) × 100.

### 2.13. Cell Cycle and Flow Cytometry Analyses

rbVFEs were seeded under four different culture conditions at a density of 10 K/cm^2^. After 36 h of seeding, in order to synchronize the cells, the culture medium was suctioned, and a fresh medium deprived of all three growth factors (ROCKi, EGF and STO-CM) was added to all four groups; the cells were incubated for 4 h in a 37 °C incubator with 5% CO_2_. Subsequently, the respective growth factors were replenished, and the cells were allowed to cycle for the following 6 h. The cells were trypsinized, collected, passed through a 40-μm cell strainer, and then fixed with 70% cold ethanol at 4 °C overnight. The cells were permeabilized using 0.2% Triton X-100 in 1% BSA for 30 min at RT followed by a single wash with 1× PBS. Subsequently, the cells were pelleted by centrifugation at 1200 rpm for 5 min at RT and incubated with 0.5 μL FxCycle™ Violet Stain (Cat. # F10347, Thermo Fisher Scientific, Waltham, MA, USA), which was added per 1 × 10^6^ cells in 1 mL of PBS and incubated for 30 min on ice. Cells in the S-phase were quantified using FACS analysis (FACS Canto II, Becton Dickinson and Company, Franklin Lakes, NJ, USA) at the Soonchunhyang Biomedical Research Core Facility of the Korea Basic Science Institute (KBSI).

### 2.14. Quantitative Real-Time PCR (qPCR)

To assess gene expression, rbVFEs were seeded under the respective culture conditions at a density of 10 K/cm^2^. After 72 h, the cells were trypsinized, washed, and then stored in TRIzol reagent (Cat. # 15596026, Invitrogen, Carlsbad, CA, USA) at −80 °C until RNA extraction. Following the manufacturer’s instructions, RNA was extracted, and RNA was measured using a NanoPhotometer N60 (Implen Scientific Inc., Munich, Germany). Reverse transcription was performed using ReverTra Ace^®^ qPCR RT Master Mix (Cat. # FSQ-301, TOYOBO, Osaka, Japan) to generate cDNA. Total RNA (1 μg) was used as a template for the 10 μL reaction. qPCR was performed in triplicate using SYBR^®^ Green Realtime PCR Master Mix (Cat. # F0924K; TOYOBO, Osaka, Japan); primer sequences are listed in Supplementary Table S1. The qPCR amplification was performed using a QuantStudio 1 Real-Time PCR System (Applied Biosystems™, Foster City, CA, USA). Gene expression levels were normalized to glyceraldehyde-3-phosphate dehydrogenase (GAPDH), which served as an internal reference. Data were analyzed using the 2^−ΔΔCt^ method, and the expression of each gene of interest was compared to that of the control sample. The results were presented as relative fold changes in gene expression.

### 2.15. Preparation of Polyacrylamide (PAA) Gel

We used a 1.8 kPa PAA hydrogel to replicate the physiological stiffness of the VFs. PAA hydrogels were prepared in glass-bottomed dishes (Cat. # 101350, SPL Life Sciences, Pocheon, Republic of Korea) following the established protocols [32,33]. Briefly, the glass surface was rendered hydrophilic by flame-treating with a Bunsen burner for 1–2 s, followed by treatment with 0.1 M sodium hydroxide (Cat. # 39155S0350, Tokyo Chemical Industry, Tokyo, Japan) for 5 min at room temperature (RT). The surface was suctioned, air-dried, treated with 3-aminopropyltrimethoxysilane (Silane, Cat. # 28177-500ML, Sigma-Aldrich, St. Louis, MO, USA) for 90 s, rinsed with distilled water, then incubated with 0.5% glutaraldehyde (Cat. # G5882, Sigma-Aldrich, St. Louis, MO, USA) for 30 min at RT. The dishes were washed, air-dried, stored in a desiccator, and used within 2 months of preparation.

The 1.8 kPa gel premix was prepared using 40% acrylamide (Cat. # 161-0140, Bio-Rad, Hercules, CA, USA) and 2% bis-acrylamide solution (Bis Solution, Cat. # 161-0142, Bio-Rad, Hercules, CA, USA) mixed with distilled water to a final AA/bis ratio of 5%:0.06%. HEPES (1M) (Cat. # 15630-080, Gibco™, Thermo Fisher Scientific, MA, USA) was added to the premix to balance the pH. Gel polymerization was initiated by adding ammonium persulfate (APS, 0.6% *w*/*v* in DI water, Cat. # AMP001, BioShop, Burlington, ON, Canada) and N,N,N′,N′-tetramethylethylenediamine (TEMED, 0.4% *v*/*v* in DI water, Cat. #TEM001, BioShop, Burlington, ON, Canada). For the cellular force analysis, red fluorescent beads (0.01%, ex/em = 580/605 nm; dia. = 0.5 μm) (Cat. # F8812, Invitrogen, Waltham, MA, USA)) were added to the PAAm gel.

Fifteen microliters of gel premix were applied to the activated glass surface, overlaid with an untreated 18 mm coverslip (Cat. # 0111580, Marienfeld, Königshofen, Germany), and allowed to polymerize for 1 h at RT. After polymerization, distilled water was added to the dish, and the coverslip was gently removed using a razor blade. The gel was then washed thoroughly with distilled water.

To promote cell attachment, the bi-functional cross-linker N-sulfosuccinimidyl-6-[4′-azido-2′-nitrophenylamino] hexanoate (Sulfo-SANPAH, 0.5 mg/mL, Cat. # 22589, Thermo Scientific, Mississauga, ON, Canada) was added to the gel and crosslinked under UV light (365 nm, Cat. # UVP95004206, Blak-Ray^®^XX-15L, Upland, CA, USA) for 10 min. Sulfo-SANPAH crosslinking was performed twice, and the gels were then washed with water. Collagen type I (100 μg/mL; cat. # 354236, Corning, Corning, NY, USA) was conjugated to the Sulfo-SANPAH-coated PAAm gel and incubated overnight at 4 °C. The unattached collagen was washed off, and the gels were sterilized under UV light (254 nm, Cat. # 95004209, Mineralight^®^ XX-15S, Upland, CA, USA) for 10 min. Finally, the gels were preconditioned by equilibrating them with the respective cell culture medium at 37 °C in a 5% CO_2_ environment for a minimum of 10 min before cell seeding.

### 2.16. IF Staining Images Analyses

The rbVFEs were seeded under four culture conditions at a density of 10,000 cells/cm^2^ and cultured for at least one passage before immunofluorescence (IF) analysis. Cells were then reseeded at 5000 cells/cm^2^ on glass-bottom dishes (SPL Flux Bottom Dish, Cat. # 211350, SPL Life Sciences, Pocheon-si, Gyeonggi-do, Republic of Korea). After 24 h, the cells were fixed and processed for one of three staining protocols:

- Set 1: anti-YAP (clone 63.7, Cat. # sc-101199, Santa Cruz Biotechnology, Dallas, TX, USA) and anti-FAK (phospho Y397, clone EP2160Y, Cat. # ab81298, Abcam, Cambridge, UK)
- Set 2: Paxillin monoclonal antibody (clone 5H11, Cat. #MA5-13356, Thermo Fisher Scientific, dilution 1:200) and anti-p63 (clone EPR5701, Cat. # ab124762, Abcam, dilution 1:200)
- Set 3: anti-BCL-2 (clone 124, Cat. # sc-7382, Santa Cruz Biotechnology, Dallas, TX, USA) and anti-cytokeratin 14 (clone SP53, Cat. # ab119695, Abcam, dilution 1:200)

All sets included counterstaining with Alexa Fluor™ 488 Phalloidin (Cat. # A12379, Invitrogen, dilution 1:400) and Hoechst 33342 (Cat. # H21492, Thermo Fisher Scientific, dilution 1:500). Images were acquired using the EVOS™ M7000 Imaging System (Thermo Fisher Scientific) at 0.31 µm/pixel resolution.

The images were analyzed using a custom MATLAB program (MathWorks, Natick, MA, USA; version R2023b) to minimize the subjective determination of threshold values, thereby reducing variability and experimental errors, as detailed in a previous study [34]. Briefly, histogram-based thresholding algorithm (HTA) involved the following steps: (1) background noise elimination by subtracting 0.5% of the minimum values in the histogram; (2) drawing a region of interest (ROI) to confine cell analysis; (3) determining the absolute threshold using a combination of means (m) and standard deviations (σ) of intensity, along with a relative threshold value (α), where approximately 5% of maximum intensity was used as a threshold; and (4) applying minimum and maximum size filters of 1.5 μm^2^ and 30 μm^2^, respectively, to eliminate the noise from protein fragments and aggregates. Following binarization using HTA, morphological and intensity parameters of FAs were extracted.

These parameters included the area, centroid, maximum axis length, elongation, and circularity of individual FAs according to the method described for cellular morphology analysis. The total intensity was obtained by summing the pixel values within the FA boundaries, and the mean intensity was calculated by dividing the total intensity by the FA area. For improved visualization, images were re-acquired using Leica Microsystems. THUNDER Imager 3D Cell (Leica Microsystems, Wetzlar, Germany). The percentage of P63-positive cells was calculated as the total number of P63-stained cells divided by the total number of DAPI-stained cells, multiplied by 100. P63 intensity was quantified using MATLAB software, with an intensity threshold of 500 considered P63-positive for rbVFEs cultured on TCP and an intensity threshold of 1000 considered P63-positive for rbVFEs cultured on 1.8 kPa gel.

### 2.17. Cellular Forces Analysis

The rbVFEs were seeded under 4 different culture conditions at a density of 10 K/cm^2^ and cultured for at least one passage before preparation for cellular force analysis. rbVFEs in each group were seeded under their respective culture conditions at a density of 5 K/cm^2^ in 1.8 kPa PAA gel containing red fluorescent beads. After 24 h of seeding, the rbVFEs were stained with CellTracker™ Fluorescent Probes, Green (Cat. # C7025, Thermo Fisher Scientific, Waltham, MA, USA) for 30 min in a 37 °C incubator with 5% CO_2._ Images were captured using a confocal microscope (LSM 710; Carl Zeiss, Oberkochen, Germany) at the Soonchunhyang Biomedical Research Core Facility of the Korea Basic Science Institute (KBSI). Traction force microscopy (TFM) measurements were performed as described by Hur et al. [35]. The image pixel size was set to 0.35 μm/pixel. Bead displacements were calculated using the particle image velocimetry (PIV) method in MATLAB (MathWorks) by comparing images in a null force state (absence of cells) and force state (presence of cells) [36]. To achieve null-force state, 1 mL of 10% *w*/*v* sodium lauryl sulfate (SDS, 196-08675, Wako Chemicals, Richmond, VA, USA) and 1% *v*/*v* Triton X-100 (Cat. # TRX777.500, Bioshop, Burlington, ON, Canada) in distilled water were used to remove the cells. The finite element method (FEM) was employed to solve partial differential equations (PDEs) using the Abaqus software (version 6.14; Dassault Systèmes^®^, Vélizy-Villacoublay, France). Subsequently, traction stress vectors in the X- and Y-directions were derived from the stress tensor obtained through the FEM analysis.

### 2.18. Statistical Analysis

Data were statistically analyzed using GraphPad Prism (version 10.4.0, Graph Pad Software Inc., San Diego, CA, USA). Data was presented as the mean ± standard deviation (SD), and statistical significance was assessed using one-way analysis of variance (ANOVA) or two-way ANOVA followed by Tukey’s multiple comparison test. * *p* < 0.05, ** *p* < 0.01, *** *p* < 0.001.

## 3. Results

### 3.1. Rabbit VF Epithelial Cell Isolation, Purification, Characterization, and Maintenance

**Figure 1:**
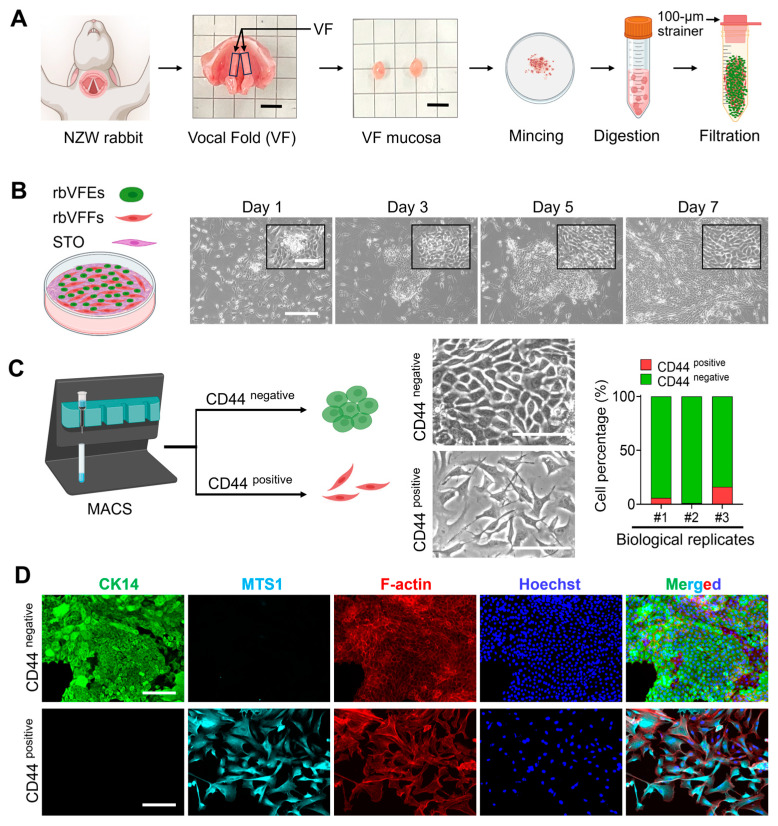
Rabbit VF epithelial cell isolation, purification, and characterization. (**A**) New Zealand White breeder (NZW) rabbit, vocal fold harvested from NZW rabbit (scale bar = 5 mm), mucosa cut out from the vocal fold (scale bar = 5 mm) and cell isolation procedure (images are created with Biorender). (**B**) Time-dependent images of VF cell colony growth. Scale bar = 300 µm and 100 µm (cropped image). (**C**) CD44 labeling, magnetic activated cell sorting of VF-derived cells, phase-contrast images, and quantification of CD44-positive and CD44-negative cells. Scale bar = 100 µm. (**D**) Characterization of CD44-sorted cells with epithelial and fibroblast-oriented markers. Scale bar = 150 µm. Abbreviations: NZW, New Zealand White; VF, vocal fold; rbVFEs, rabbit VF epithelial cells; rbVFFs, rabbit VF fibroblasts; CK14, cytokeratin 14; MTS1, metastasin.

In primary cultures, rbVFEs formed colonies within 24 h. These colonies exhibited a cobblestone appearance and formed borders distinct from the surrounding feeder cells. The colonies reached confluence within 1 week of the initial plating (Figure 1B). However, overgrowth of rabbit VF fibroblasts (rbVFFs) was observed, which required additional purification steps. We performed indirect MACS by labeling cells with a CD44 antibody. After sorting, the average percentages of CD44-positive and CD44-negative cells were 7.7% and 92.28%, respectively, in three biological specimens (Figure 1C). When the CD44-positive and CD44-negative cell fractions were seeded in epithelial cell-oriented media, CD44-positive cells exhibited a mesenchymal phenotype, whereas CD44-negative cells displayed a cuboidal epithelial morphology (Figure 1C). To characterize MACS-sorted, rabbit VF-derived epithelial cells and fibroblasts, we performed co-staining with CK14 and Mts1. Immunostaining revealed that CD44-positive cells expressed Mts1 but lacked CK14, whereas CD44-negative cells expressed cytokeratin 14 without Mts1 expression (Figure 1D). These results indicate the successful purification of rbVFEs. We replaced STO feeder with STO-conditioned media (STO-CM) and observed higher rbVFE proliferation with STO-CM rather than the STO feeder itself as depicted by a cell proliferation assay (Figure S3A,C) and quantification of rbVFEs in s-phase with Edu assay (Figure S3B,D). Thus, after the initial cell isolation and MACS-based purification, we transitioned to using STO-CM. Our culture method successfully supported the long-term expansion of rbVFEs (Figure S4A,B). Despite the expansion being performed with rbVFEs sorted solely using the differential adhesion technique (Figure S2A,B), the rbVFEs formed an almost pure population, as evidenced by the confluent CK14-positive cells (Figure S2C). Several studies have relied on adhesion-based purification and immunocytochemistry with epithelial markers, such as cytokeratin, to characterize VFE cells [4,37]. Eventually, the MACS-sorted rbVFEs at passages 3–7 were used for experimental purposes.

### 3.2. Role of ROCKi, EGF, and STO-CM in Driving Proliferation of rbVFEs

**Figure 2:**
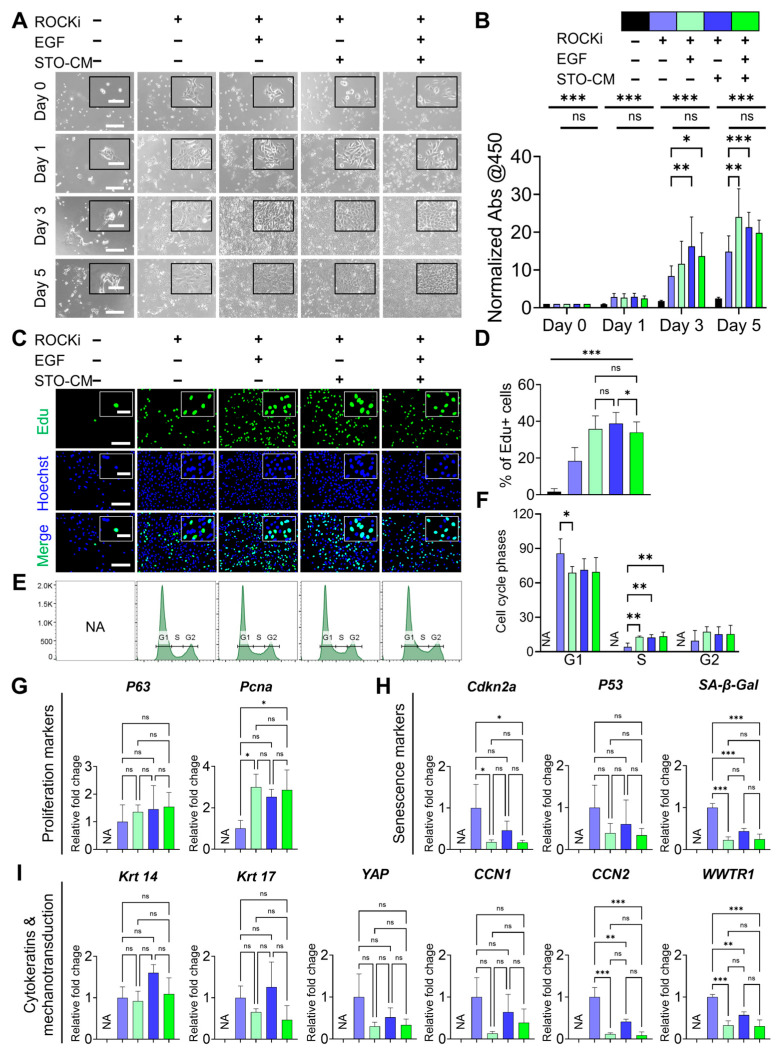
Role of ROCKi, EGF, and STO-CM in supporting proliferation of rbVFEs. (**A**) Time-dependent culture images of rbVFEs. Scale bar = 300 µm and 100 µm (cropped image). (**B**) Quantification of rbVFEs daily proliferation with cells via assay, 1st group, *n* = 5 (from 1 biological specimen), and 2nd to 5th groups, *n* = 16, 16, 16, 16 (from 3 biological specimens). Data are presented as the mean ± SD, * *p* < 0.05, ** *p* < 0.01, *** *p* < 0.001 using two-way ANOVA test followed by Tukey’s multiple comparisons test. (**C**) Click iT Edu images for visualization of proliferating rbVFEs. Scale bar = 150 µm and 50 µm (cropped image). (**D**) Quantification of rbVFEs in S-phase, 1st–5th group, *n* = 807, 4461, 4710, 4946 (from 3 biological specimens). (**E**,**F**) Cell cycle analysis conducted using FxCycle™ staining, 1st group, *n* = 0, 2nd to 5th group, *n* = 700 K, 600 K, 700 K, 700 K (from 2 biological specimens). (**G**) Gene expression of proliferation markers (P63 and Pcna). (**H**) Gene expression of senescence-related markers (Cdkn2a, P53 and SA-β-Gal). (**I**) Gene expression of cytokeratins (CK14 and CK 17) and mechanotransduction-related markers (YAP, CCN1, CCN2, WWTR1). *n* = 3 biological specimens for qPCR. (**D**,**F**–**I**) Data are presented as the mean ± SD, ns, *p* > 0.05; * *p* < 0.05; ** *p* < 0.01; *** *p* < 0.001 using one-way ANOVA test followed by Tukey’s multiple comparisons test. Abbreviations: ROCKi, ROCK inhibitor (Y-27682); CM, conditioned media; Abs, absorbance; Pcna, proliferating cell nuclear antigen; Cdkn2a, cyclin-dependent kinase inhibitor 2A; SA-β-Gal, senescence-associated beta galactosidase; Krt, Cytokeratin; YAP, Yes-associated protein; CCN1, cellular communication network factor 1; CCN2, cellular communication network factor 2; WWTR, WW domain-containing transcription regulator; NA, not applicable.

The proliferation of rbVFEs was highly dependent on the presence of ROCKi, EGF, and STO-conditioned media (STO-CM). In the absence of these three growth factors, rbVFEs neither adhered properly to the tissue culture plates (TCP) nor proliferated. Supplementation with ROCKi alone enabled rbVFEs to attach to TCP; however, their proliferation remained minimal. The addition of EGF or STO-CM to ROCKi-containing media significantly enhanced rbVFE proliferation (Figure 2A,B). In the cell proliferation assay, by day 3, EGF with ROCKi and STO-CM with ROCKi increased the absorbance values by 1.3-fold and 2-fold, respectively, compared with the ROCKi-only group. However, the combination of all three growth factors together did not provide any additional benefits, resulting in only a 1.6-fold increase. By day 5, EGF with ROCKi increased proliferation by 1.6-fold; STO-CM with ROCKi increased proliferation by 1.4-fold; and the combination of all three factors led to a 1.3-fold increase. The observed decline in proliferation in the STO-CM with the ROCKi group by day 5 may be attributed to confluence, as cell proliferation typically plateaus when cultures reach confluency [38]. Proliferating rbVFEs under the influence of these growth factors were also visualized using EdU imaging (Figure 2C,D). Consistent with our proliferation pattern, the percentage of EdU-positive cells was less than 2% in the absence of ROCKi, EGF, and STO-CM. Supplementation with ROCKi increased the percentage of EdU-positive cells to only 18%. Compared to the ROCKi-only group, EGF with ROCKi, STO-CM with ROCKi, and all three growth factors together increased EdU-positive cells to 36%, 39%, and 34%, respectively. Because rbVFEs failed to proliferate and their condition deteriorated in the absence of ROCKi, EGF, and STO-CM, this group was excluded from further experiments. The FACS sorting of rbVFEs in different phases of the cell cycle showed a trend similar to that of EdU. With ROCKi only, 86% of the cells remained in the G1 phase, and only 4% of the cells entered the S-phase. EGF with ROCKi, STO-CM with ROCKi, and all three growth factors together reduced the percentage of cells in the G1 phase to 68.87%, 71.34%, and 69.56% and increased the percentage of cells entering the S-phase to 12.95%, 12.44%, and 13.51%, respectively (Figure 2E,F). To further assess the effect of ROCKi, EGF and STO-CM, we examined the mRNA expression of proliferation and senescence-related markers. The ROCKi-only group expressed low levels of proliferation markers PCNA and P63 (Figure 2G) and significantly high levels of senescence markers CDKN2A, P53, and Senescence-associated beta galactosidase (SA-β-Gal) (Figure 2H). EGF with ROCKi, STO-CM with ROCKi, and the group of all three growth factors together enhanced the expression of PCNA and P63 and downregulated the senescence marker CDKN2A, p53, and SA-β-Gal. Cytokeratin 14 and 17 levels increased in the STO-CM with the ROCKi-only group (Figure 2I). Additionally, we analyzed the mechanotransduction genes Yes-associated protein (YAP), Cellular communication network factor 1 (CCN1), Cellular communication network factor 2 (CCN2), and WW domain-containing transcription regulator (WWTR). All of these genes were upregulated in the ROCKi-only group (Figure 2I).

### 3.3. Role of ROCKi, EGF, and STO-CM in Modulating YAP Activation and Adhesion Dynamics and Cellular Senescence

**Figure 3:**
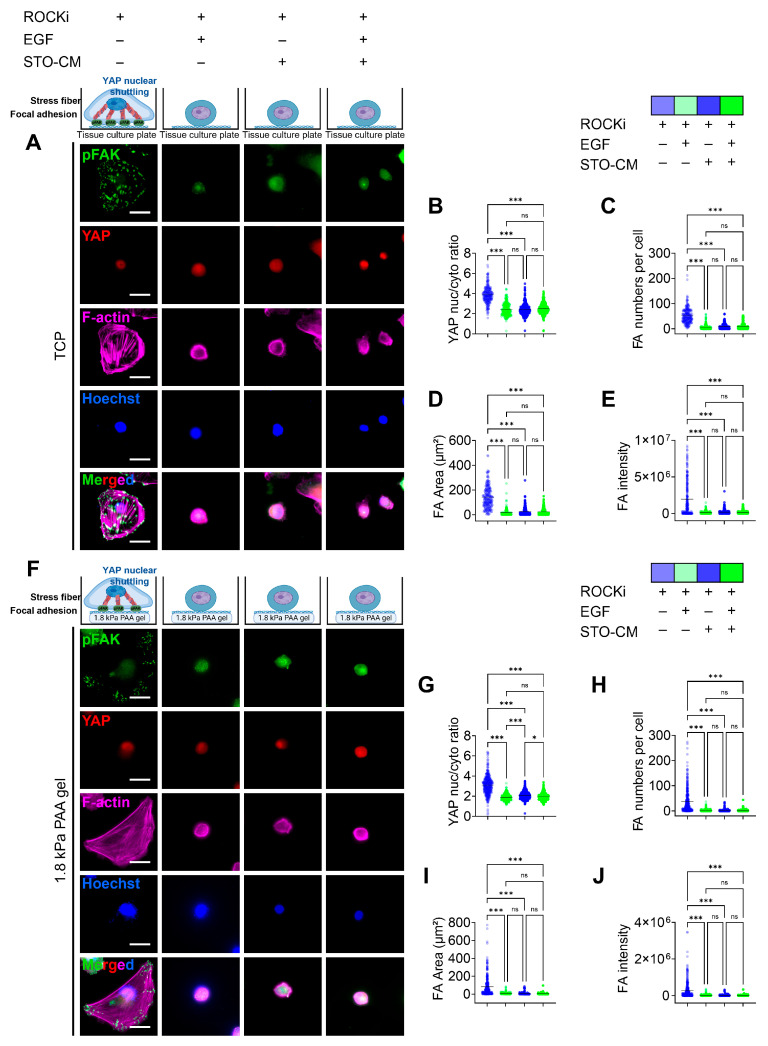
Role of ROCKi, EGF, and STO-CM in driving YAP nuclear translocation and phosphorylated focal adhesion kinase (pFAK)-based focal adhesion (FA) formation. (**A**) IF staining for YAP and pFAK for rbVFEs cultured in different conditions on TCP. Scale bar = 25 µm. (**B**) YAP nuclear translocation, (**C**) number of pFAK-based focal adhesion, (**D**) total sum of FA area formed per cell, (**E**) intensity of pFAK-based FA (1st–4th group, *n* = 241, 207, 273, 270, respectively). (**F**) IF staining for YAP and pFAK for rbVFEs cultured in different conditions on 1.8 kPa gel. Scale bar = 25 µm. (**G**) YAP nuclear translocation, (**H**) number of pFAK-based focal adhesion per cell, (**I**) total sum of FA area formed per cell, (**J**) intensity of pFAK-based FA (1st–4th group, *n* = 369, 329, 359, 351, respectively). Data are plotted as individual points, with the line representing the mean value, ns, *p* > 0.05; * *p* < 0.05; *** *p* < 0.001 using one-way ANOVA test followed by Tukey’s multiple comparisons test. Abbreviations: TCP, tissue culture plastic.

We observed changes in the morphological features of rbVFEs under various culture conditions. Specifically, we noted increased cell spreading beyond average size, accompanied by Yes-associated protein (YAP) nuclear translocation, focal adhesion kinase (FAK) phosphorylation to form focal adhesions, and stress actin fiber formation in the ROCKi-only group on both TCP (Figure 3A) and 1.8 kPa PAA gel (Figure 3F). In TCP, the ROCKi-only group had a YAP nucleus-to-cytoplasm ratio of 3.876 ± 0.85, while ROCKi+ EGF, ROCKi + STO-CM, and all three growth factors together had YAP nucleus-to-cytoplasm ratios of 2.431 ± 0.62, 2.437 ± 0.58, 2.49 ± 0.52 (Figure 3B). Similarly, in 1.8 kPa PAA gel, the ROCKi-only group had a YAP nucleus-to-cytoplasm ratio of 3.1 ± 0.93, while ROCKi+ EGF, ROCKi + STO-CM, and all three growth factors together had YAP nucleus-to-cytoplasm ratios of 1.89 ± 0.32, 2.09 ± 0.39, 1.96 ± 0.35 (Figure 3G). pFAK-based focal adhesion was higher in number, total area, and intensity in the ROCKi-only group in both TCP (Figure 3C–E) and 1.8 kPa gel (Figure 3H–J). We have added the MATLAB-processed images in Figure S5 for clarity.

### 3.4. Correlation Between Paxillin-Based Focal Adhesion Dynamics and Self-Renewal Marker (p63) Expression

**Figure 4:**
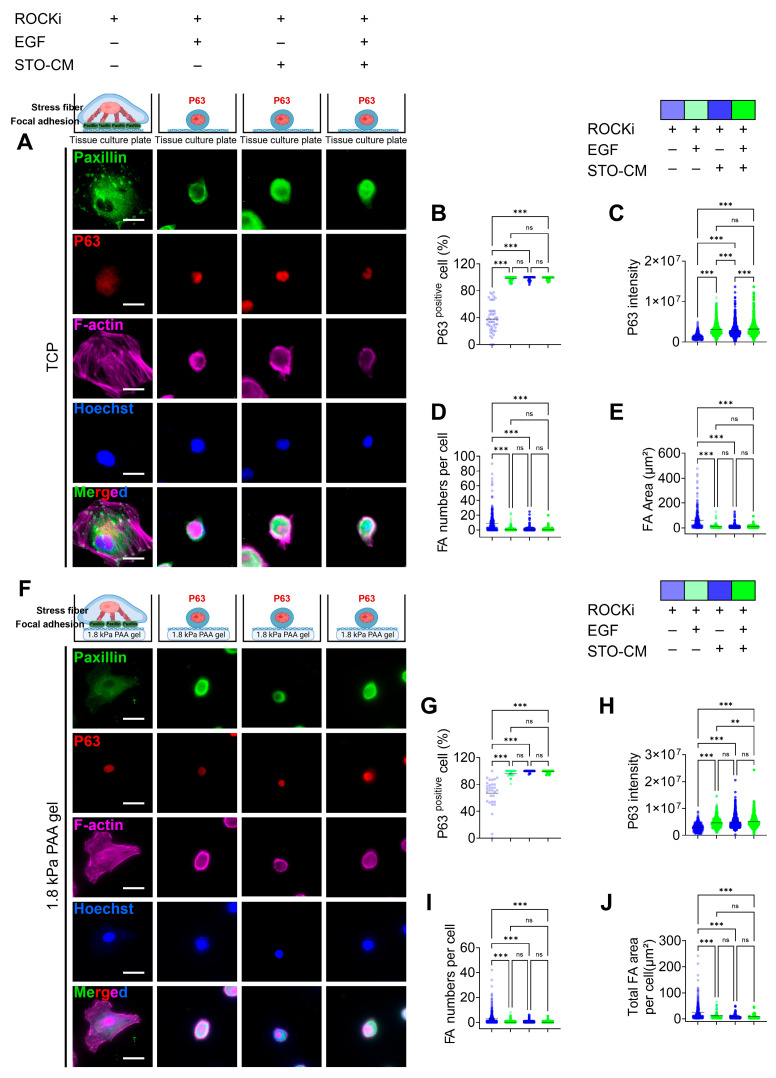
Correlation between paxillin-based focal adhesion formation and self-renewal marker (p63) expression. (**A**) Immunofluorescence staining for p63, paxillin, and F-actin in different culture condition tissues when cultured on TCP. Scale bar = 25 µm. (**B**) p63-positive nucleus percentage and (**C**) p63 intensity analysis (1st–4th group, *n* = 491, 1249, 1173, 1296, respectively). (**D**,**E**) Paxillin-based focal adhesion (FA) analysis (FA number per cell and total sum of FA area per cell) (1st–4th group, *n* = 356, 546, 853, 698, respectively). (**F**) Immunofluorescence staining for p63 and paxillin and F-actin in different culture conditions, when cultured on gel. Scale bar = 25 µm. (**G**) p63-positive nucleus percentage and (**H**) p63 intensity analysis (1st–4th group, *n* = 423, 457, 570, 590, respectively). (**I**,**J**) Paxillin-based focal adhesion (FA) analysis (FA number per cell and total sum of FA area per cell) (1st–4th group, *n* = 477, 736, 901, 832, respectively). Data are plotted as individual points, with the line representing the mean value, ns, *p* > 0.05; ** *p* < 0.01; *** *p* < 0.001 using one-way ANOVA test followed by Tukey’s multiple comparisons test.

Similarly to pFAK expression, the ROCKi-only group or non-proliferative group showed significant focal adhesion formation, whereas all other three actively proliferating groups had uniformly distributed paxillin throughout the cell (Figure 4A,F). The number of paxillin-based FA and sum of area of FA was significantly higher in rbVFEs in the ROCKi-only group in both TCP (Figure 4D,E) and 1.8 kPa gel (Figure 4I,J). At the protein level, using ROCKi alone, the percentage of the self-renewal marker (p63)-positive cells was 37.55 ± 20.69. Incorporation of EGF, STO-CM, or both increased this percentage to 97.46 ± 3.5, 98.44 ± 2.82, and 98.67 ± 2.1, respectively (Figure 4B). Similarly, the mean P63 intensity was significantly increased by the incorporation of EGF, STO-CM, or both (Figure 4C). These results indicate that ROCKi should be supplemented with either EGF or STO-CM to promote the self-renewal of rbVFEs. Similarly, rbVFEs cultured on 1.8 kPa PAA gel exhibited a similar trend. Using ROCKi alone, the percentage of the self-renewal marker (p63)-positive cells was 66.87 ± 22.31. Incorporation of EGF, STO-CM, or both increased this percentage to 96 ± 5.12, 99.4 ± 1.5, and 98.71 ± 2.17, respectively (Figure 4G,H). The representative MATLAB-processed images used for P63 intensity calculation are shown in Figure S6 for clarity.

### 3.5. Effect of ROCKi, EGF, and STO-CM in Cytoskeletal Protein Remodeling and Apoptosis Marker Expression

**Figure 5:**
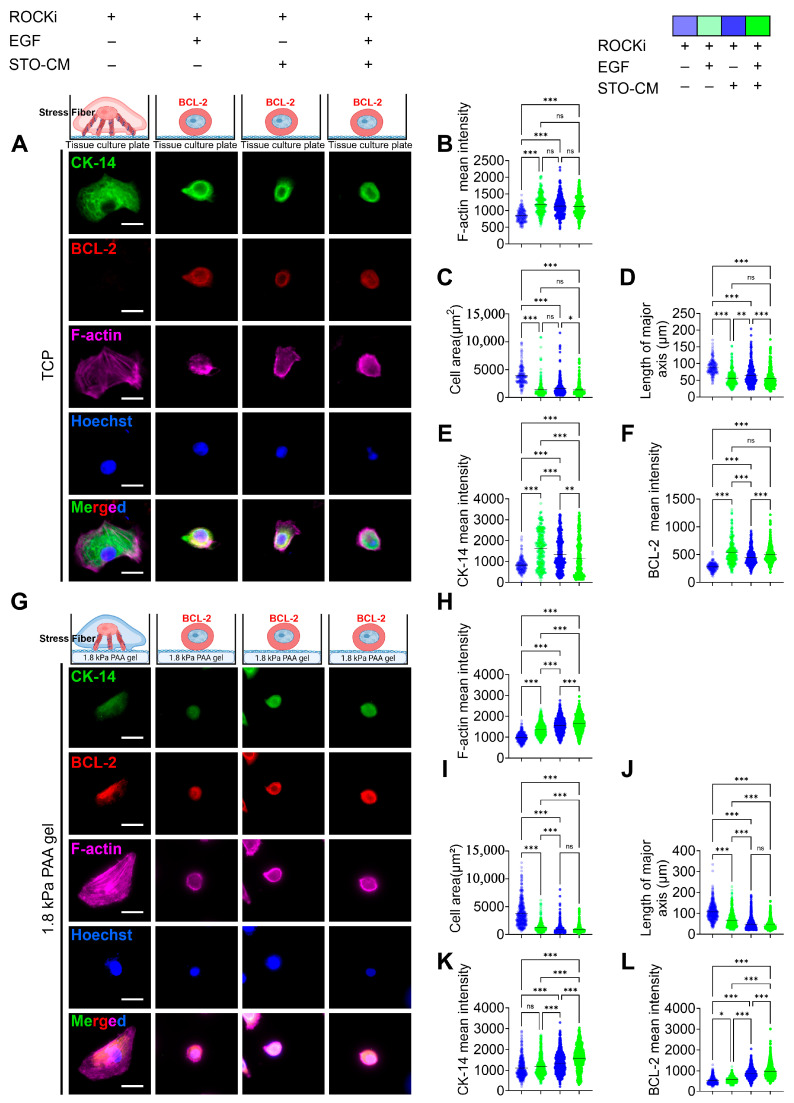
Cytoskeletal remodeling and anti-apoptosis marker (BCL-2) expression under the influence of ROCKi, EGF, and STO-CM. (**A**) Immunofluorescence staining for CK14, BCL-2, and F-actin in different culture conditions on tissue culture plate. Scale bar = 25 µm. (**B**) F-actin mean intensity analysis. (**C**) Cell area. (**D**) Length of major axis of cell. (**E**) CK-14 mean intensity analysis. (**F**) BCL-2 intensity analysis (1st–4th group, *n* = 176, 260, 354, 353, respectively). (**G**) Immunofluorescence staining for CK14, BCL-2, and F-actin in different culture conditions on 1.8 kPa gel. Scale bar = 25 µm. (**H**) F-actin analysis. (**I**) Cell area. (**J**) Length of major axis of cell. (**K**) CK-14 mean intensity analysis. (**L**) BCL-2 intensity analysis (1st–4th group, *n* = 390, 520, 797, 784, respectively). Data are plotted as individual points, with the line representing the mean value, ns, *p* > 0.05; * *p* < 0.05; ** *p* < 0.01; *** *p* < 0.001 using one-way ANOVA test followed by Tukey’s multiple comparisons test.

The expression of basal cytokeratin 14 (CK-14) was greatly reduced when the cells were cultured with ROCKi alone in both TCP (Figure 5A) and 1.8 kPa PAA gel (Figure 5G). However, the groups further supplemented with EGF, STO-CM, or both showed a significantly higher expression of CK-14. Additionally, in the ROCKi-only group, actin was bundled to form prominent stress fibers, whereas in the other three groups, cortical F-actin was expressed evenly throughout the cell (Figure 3, Figure 4 and Figure 5). However, the mean F-actin intensity was significantly lower in the ROCKi-only group, suggesting that the very low levels of actin expressed in the ROCKi-only group were remodeled to generate prominent stress fibers to provide mechanical strength to the cell for survival. The ROCKi group exhibited significantly large cellular area and length of the major axis in both TCP (Figure 5C,D) and 1.8 kPa gel (Figure 5I,J). In the TCP, the mean cell area for rbVFEs cultured in the ROCKi-only group was 3903 ± 1709, while in the other three groups the mean cell area were 1375 ± 1375, 1632 ± 1340, 1361 ± 1134 for ROCKi + EGF, ROCKi + STO-CM, and ROCKI + EGF + STO-CM, respectively. Similarly, in 1.8 kPa PAA gel, the mean cell area for rbVFEs cultured in the ROCKi-only group was 3790 ± 2246, while in the other three groups the mean cell areas were 1221 ± 745, 898 ± 671, and 849.3 ± 587 for ROCKi + EGF, ROCKi + STO-CM, and ROCKI + EGF + STO-CM, respectively. The excessive spreading of rbVFEs when cultured with ROCKi-only further supports our claim that without EGF or STO-CM, rbVFEs are driven towards cellular senescence. Furthermore, the intensity of anti-apoptotic marker BCL-2 was significantly low in the ROCKi-only group in both TCP (Figure 5F) and 1.8 kPa PAA gel (Figure 5L). The representative MATLAB-processed images used for P63 intensity calculation are shown in Figure S7 for clarity.

### 3.6. Cellular Traction Forces and Intracellular Tension During Senescence or Self-Renewal

**Figure 6:**
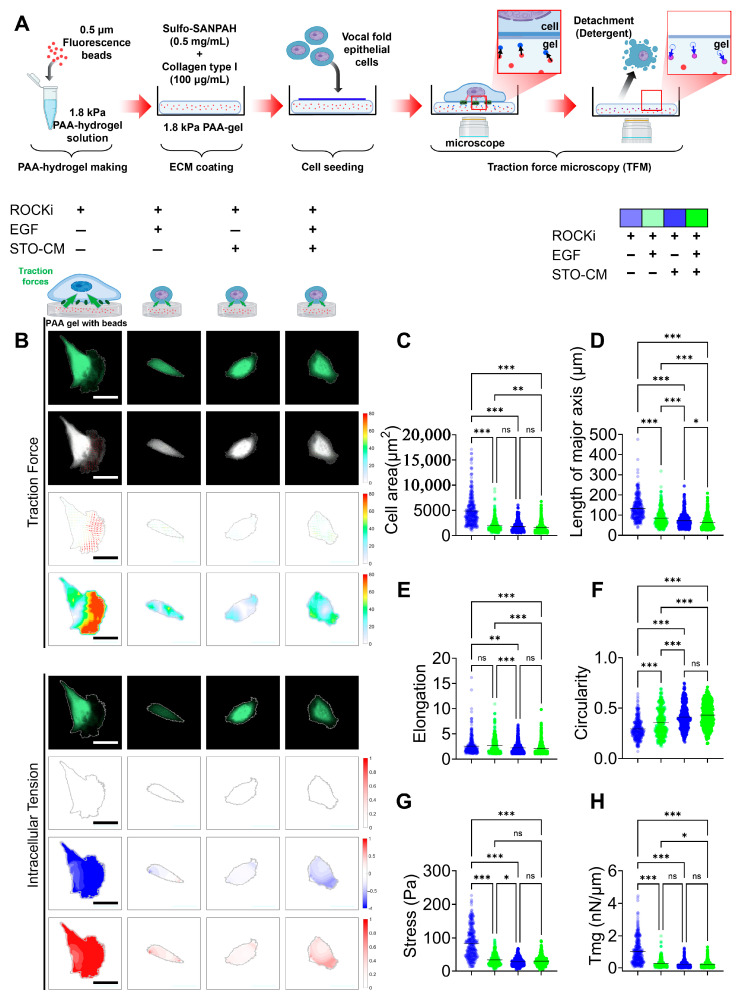
Cellular traction force and intracellular tension under the influence of ROCKi, EGF, and STO-CM. (**A**) Schematic illustration of traction force microscopy (TFM) setup. Polyacrylamide (PAA) hydrogels (1.8 kPa) embedded with fluorescent beads were conjugated with collagen type I using Sulfo-SANPAH and seeded with rabbit vocal fold epithelial cells (rbVFEs) under different culture conditions (ROCKi, EGF, and STO-CM). Bead displacement was imaged before and after cell detachment to calculate traction forces and intracellular tension. (**B**) Fluorescence images of rbVFEs and respective contour images indicating cellular traction force and intracellular tension. Arrow color represents the magnitude of traction stresses, and arrow orientation indicates their direction. Scale bar = 50 µm. (**C**) Cell area. (**D**) Length of major axis of cell. (**E**) Cell elongation. (**F**) Cell circularity. (**G**) Cellular traction force. (**H**) Intracellular tension. (1st–4th group, *n* = 337, 308, 355, 407). Data are plotted as individual points, with the line representing the mean value, ns, *p* > 0.05; * *p* < 0.05; ** *p* < 0.01; *** *p* < 0.001 using one-way ANOVA test followed by Tukey’s multiple comparisons test.

We measured both the traction force and intracellular tension generated by rbVFEs under the influence of the growth factors (ROCKi, EGF, and STO-CM) (Figure 6). Both the cellular traction force (Figure 6F) and intracellular tension (Figure 6G) were significantly increased in the ROCKi-only group, while the other three groups exhibited low cellular forces and intracellular tension. Additionally, to validate the accuracy of our force analysis method, we calculated the morphology parameters such as cellular area, length of major axis of cell, cellular elongation, and circularity (Figure 6B–E). The morphology parameters (cell area and length of major axis) aligned with our earlier results (Figure 5I,J). Since rbVFEs in ROCKi-only group exhibited senescent features, our data provides a correlation between cellular forces and epithelial cell senescence. Cellular stress was highest in the ROCKi-only (senescent) group with a mean value of 83.64 ± 44.64, while other 3 (proliferating or self-renewing) groups had mean cellular traction force of 34.18 ± 17.19, 28.33 ± 12.81, 30.31 ± 14.67 for ROCKi + EGF, ROCKi + STO-CM and ROCKi + EGF + STO-CM, respectively. Similarly, intracellular tension was highest in the ROCKi-only (senescent) group with a mean value of 1 ± 0.78, while the other three (proliferating or self-renewing) groups had a mean cellular traction force of 0.29 ± 0.28, 0.21 ± 0.19, and 0.2 ± 0.09 for ROCKi + EGF, ROCKi + STO-CM and ROCKi + EGF + STO-CM, respectively. We observed a proliferation trend in the order ROCKi-only < ROCKi + EGF < ROCKi + STO-CM (Figure 2A–D), and we observed cellular forces (both traction stress and intracellular tension) in the order ROCKi-only > ROCKi + EGF > ROCKi + STO-CM (Figure 6F,G). These results strongly suggest the correlation between cellular forces and rbVFE proliferation, and probably self-renewal too.

## 4. Discussion

Despite several attempts using different culture medium formulations and methods across various species, no study has successfully supported VFE proliferation beyond passage four [2,3,4,5]. Stromal overgrowth is a major challenge in establishing primary epithelial cultures [39] and has been reported during VF epithelial cell establishment [4,5]. Mts1 is a fibroblast-specific marker, expressed exclusively in fibroblasts or mesenchymal cells but absent in epithelial cells [40]. Conversely, CK14 is a well-established marker of stratified squamous epithelium [41]. While fibroblasts can initially serve as feeder layers, they can often be replaced by conditioned media once the epithelial culture is established [21]. Mizuta et al. [4] previously attempted to culture rbVFEs using feeder cells (3T3-Swiss Albino (ATCC CCL^®^-92™) and media with EGF (10 ng/mL), but the cells failed to proliferate beyond passage four [4]. Given that their culture media lacked the ROCKi, we speculated that ROCKi may be a crucial factor supporting the expansion of rbVFEs. Our study highlights that ROCKi play a critical role in supporting rbVFE expansion. While Gao et al. showed ROCKi enhance adhesion and survival of human embryonic stem cells (hESCs) [42], their inhibition of ROCK1/2 impedes cell contractility, and can eventually drive cells towards multi-stage cell cycle arrest and cellular senescence [14]. This likely explains why our ROCKi-only group exhibited reduced proliferation, low EdU incorporation, and fewer cells transitioning from the G1 to S phase. Expanding epithelial cells under feeder-free conditions often faces barriers, particularly CDKN2A-dependent cell cycle arrest [43]. We observed that supplementing ROCKi-containing media with EGF or STO-CM reduced CDKN2A expression, enhancing proliferative capacity and suggesting a potential mechanism by which these factors support epithelial self-renewal. Notably, STO-CM showed the strongest proliferative effect, likely due to its mixture of soluble paracrine factors and extracellular matrix components. While we could not pinpoint the exact STO-CM components driving rbVFE proliferation, their combined effect with ROCKi appears synergistic.

Our data indicate that combining ROCK inhibition, EGF, and STO-conditioned medium (STO-CM) does not produce additive effects compared with pairwise conditions (ROCKi + EGF or ROCKi + STO-CM) and is slightly diminished. Similar observations have been reported in other systems, where the addition of growth factors to an already optimal condition failed to enhance benefit [44,45]. For instance, Krawczyk et al. demonstrated that in conditional reprogramming (CR) cultures of neuroblastoma cells, ROCKi alone supported optimal proliferation, whereas the addition of a neurotrophic factor had no effect, while the addition of corticosterone and J2 feeder cells were inhibitory [44]. Blahnova et al. [45] observed that combining multiple growth factors with overlapping functions—TGF-β, bFGF, IGF-1, HGF, and VEGF—did not enhance metabolic activity or alkaline phosphatase (ALP) activity beyond that achieved by combination of TGF- and bFGF. These outcomes likely reflect pathway saturation or redundancy due to the convergence of signaling cascades, although the precise mechanisms remain to be elucidated.

STO-CM contains multiple abundant growth factors and cytokines, including hepatocyte growth factor (HGF), stem cell factor (SCF), activin A, insulin-like growth factors 1 and 2 (IGF-1/2), IGF-binding protein 6 (IGFBP-6), macrophage colony-stimulating factor (CSF-1), pigment epithelium-derived factor (PEDF), and interferon gamma (IFN-γ) [24] which complicates identification of the specific contributors to the observed effects. Moreover, EGF is known to exhibit biphasic signaling, stimulating proliferation at low concentrations but inhibiting growth at higher doses [46]. The presence of diverse factors in STO-CM may have influenced EGF signaling through pathway redundancy or crosstalk, as many growth factor pathways converge on shared downstream effectors such as MAPK/ERK and PI3K/AKT. This overlap could lead to partial pathway saturation or modulation of receptor activity, which may reduce the responsiveness to exogenously added EGF.

Mechanotransduction pathways, particularly those involving YAP, CCN1, CCN2, and WWTR, were upregulated in the ROCKi-only group, suggesting their involvement in rbVFE proliferation and senescence. The ability of cells to perceive their ECM and spread is associated with the nuclear translocation of the Hippo pathway effectors YAP and TAZ [47]. YAP/TAZ nuclear activity is correlated with the stability of the actin cytoskeleton and cell tension, which are partially controlled by the Rho GTPase pathways [48]. Previous studies have shown that YAP plays an essential role in intestinal stem cell self-renewal and tissue regeneration [49]. YAP is a well-established mediator of cellular mechanosensing and mechanotransduction and exhibits increased nuclear translocation and activation in response to mechanical tension [50]. Notably, YAP nuclear translocation is significantly enhanced during ISC colony formation within stiff PEG hydrogels compared with softer PEG hydrogels [51]. Interestingly, however, in our system, increased YAP nuclear translocation correlated with senescence rather than self-renewal (Figure 3). Focal adhesions serve as the central hub linking ECM signals to cytoskeletal reorganization [52]. Senescent cells express mature focal adhesions as adaptations to the non-proliferative state [19,53]. For example, Chantachotikul et al. showed that late-passage human fibroblasts (senescent) exhibited more stress fibers and mature focal adhesions than early-passage cells [54]. Similarly, in our ROCKi-only group, we observed pronounced stress fibers and pFAK-based focal adhesions, supporting the idea that YAP activation here is linked to cellular senescence. We also used paxillin as a marker of focal adhesion (FA) formation because it is present throughout FA development and maturation [55] and observed results similar to pFAK. Additionally, cell morphology is closely associated with stress fiber dynamics [56]. The organization of stress fibers is altered in cells undergoing senescence [15,57]. The increased number of stress fibers together with prominent focal adhesion observed in the ROCKi-only group further suggests that the cells in the ROCKi-only group are likely undergoing senescence. Self-renewal is governed by the dynamic interplay between several intrinsic factors, including cell–cell and cell–matrix interactions [21]. Mechanobiologically, cells grown on stiff substrates assemble actin stress fibers [58], promote cellular spreading [59], modify adhesion properties [60], and stimulate cellular contractility [61], leading to increased cellular forces applied to the substrate [61].

**Figure 7:**
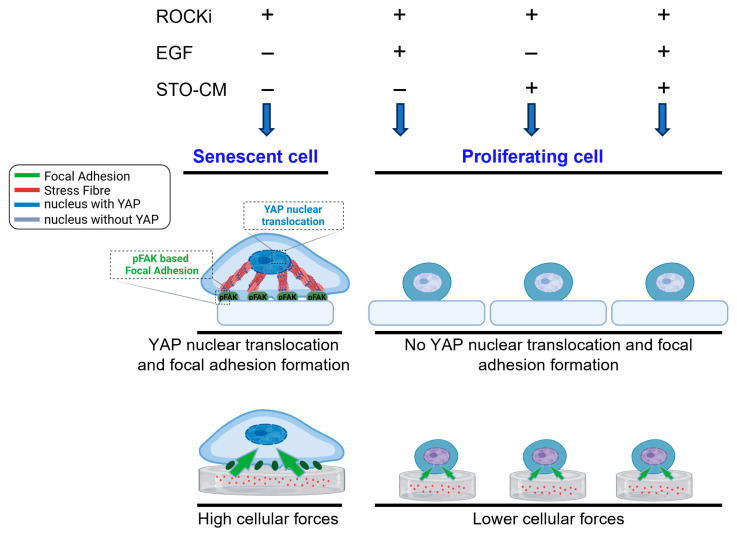
Schematic representation summarizing mechanotransduction behavior in senescent and proliferating epithelial cells.

Given the cytoskeletal changes we observed in rbVFEs, we examined how cytoskeletal remodeling affected cellular forces. Our traction force microscopy (TFM) and intracellular force microscopy (IFM) analyses showed that senescent or cells in the ROCKi-only group generated the highest cellular forces and intracellular tension, while proliferating/self-renewing groups (ROCKi + EGF, ROCKi + STO-CM and ROCKi + EGF + STO-CM) exhibited significantly lower forces. This supports the notion that reduced mechanical stress is associated with proliferation and self-renewal. Self-renewal and apoptosis are also influenced by mechanical factors, such as cell spreading and intracellular tension [62]. Prior studies suggest that a soft matrix supports self-renewal and pluripotency by generating low cell–matrix tractions [63]. For instance, Chowdhury et al. demonstrated that mESCs cultured on soft substrates maintained high levels of OCT3/4 and Nanog by reducing cell–matrix traction [63]. Similarly, Engler et al. demonstrated that MSCs tune their stiffness to match the substrate stiffness and, accordingly, undergo adipogenic (bearing low stress) or osteogenic (bearing high stress) differentiation [64]. Shoham et al. further reported that differentiated adipocytes became structurally stiffer, and the level of tensile strain increased during cell growth and maturation [65]. Cellular spreading and differentiation are correlated with the intrinsic softness of the cells, which is likely regulated by actomyosin contractility [66]. Matrix-enabled physical cues can improve the reprogramming efficiency of iPSCs derived from fibroblasts [67]. Park et al. showed that the temporal cyclic stretching of fibroblasts significantly enhanced the efficiency of induced pluripotent stem cell (iPSC) production [68]. Their study revealed that cyclic stretching enhances the biological characteristics required for pluripotency acquisition, including increased cell division and activates key mechanosensitive molecules, such as YAP, across the cytoskeletal to nuclear space [68]. Hans et al. reported that ISC expansion in stiff matrices required cytoskeletal tension and integrin-based adhesion for colony formation [51]. In a 3D culture system, Zhou Ying et al. showed that actin cytoskeleton relaxation promotes Nanog expression, a key self-renewal marker [69], while increased mechanical tension has been linked to diminished cell proliferation and enhanced differentiation, particularly when cells are cultured in stiffer environments [70]. Li et al. has claimed that cardiomyocytes respond to increased intracellular tension by altering their intercellular contacts in favor of cell–matrix interactions, leading to YAP nuclear translocation [71]. Although we did not specifically investigate the role of matrix stiffness in rbVFE self-renewal, our data reveals a relationship between cytoskeletal tension, cellular forces, proliferation, and self-renewal. By incorporating traction force microscopy (TFM) and intracellular force microscopy (IFM), we measured cellular forces and correlated them with epithelial self-renewal and senescence. As shown in Figure 7, senescent cells exert significantly higher cellular traction forces and intracellular tension compared to proliferating or self-renewing cells, which display lower forces. Figure 7 illustrates representative force maps alongside quantitative analysis, underscoring the utility of biophysical metrics to discriminate between cell states such as self-renewal and senescence. These findings demonstrate the advantage of integrating biophysical characterization to better understand the mechanisms underlying epithelial cell proliferation, senescence, and self-renewal. Our work thus lays the foundation for further studies targeting mechanotransduction pathways to optimize vocal fold epithelial cell culture and expansion.

In conclusion, our study establishes a robust reproducible culture strategy for the expansion of rabbit vocal fold (VF) epithelial cells. However, a key limitation is that all experiments were performed using VF epithelial cells derived exclusively from New Zealand White rabbits. As such, the broader applicability of this strategy to other animal models or human VF epithelial cells remains to be determined. Previous studies have highlighted species-specific differences in cellular responses to culture conditions, suggesting that media formulations optimized for animal cells may not effectively support human cell growth. For example, Katsuda et al. demonstrated that a culture strategy successful for rodent hepatocytes failed to promote proliferation in human hepatocytes [72]. These findings highlight the need for future studies aimed at developing culture strategies specifically tailored to human VF-derived epithelial cells.

Another limitation of the study is the lack of direct assessment of non-muscle myosin II (NMII) activity or localization. While direct staining for NMII isoforms could provide further mechanistic insights, our focus was on the functional outcomes of different culture conditions, including cellular proliferation, senescence, and mechanobiological behavior. These outcomes were evaluated using traction force microscopy, stress fiber and focal adhesion analyses, and YAP localization. NMII filaments are critical for cytoskeletal organization, mediating the transformation of nascent adhesions into mature focal adhesions and facilitating traction force generation [73]. NMII motor activity is required for focal adhesion assembly [74], with associated proteins recruited only in the presence of active myosin [75,76]. Intracellular tension is generally generated through the interaction of actin with phosphorylated myosin II [77], and different NMII isoforms play distinct roles: NMIIA is predominantly associated with actomyosin bundles in leading edge protrusions, while NMIIB incorporates into preformed bundles [78,79]. Upstream, NMII is regulated by Rho–ROCK signaling, with phosphorylation of heavy chains mediating adhesion maturation [80]. ROCK regulates actomyosin contractility by modulating the myosin-binding subunit of myosin phosphatase and phosphorylating myosin light chain 2 (MLC2) [81,82], as also previously demonstrated by Goeckeler et al. [83]. ROCK inhibition leads to reduced MLC phosphorylation and contractility, while NMII inhibition disrupts actin filament organization [84]. Given the central role of ROCK in NMII regulation, the observed changes in stress fiber formation, focal adhesion assembly, YAP localization, and intracellular tension in our study are likely reflective of underlying NMII activity.

Interestingly, we found that treatment with ROCK inhibitor (ROCKi) alone induced pronounced stress fiber formation, nuclear YAP localization, and the development of focal adhesions marked by paxillin and phosphorylated FAK—features indicative of increased cytoskeletal tension and potential NMII activation. However, when ROCKi was combined with either EGF or STO-conditioned media (STO-CM), these features were markedly diminished. Under these combined conditions, we observed a reduction in stress fibers and focal adhesions, cytoplasmic localization of YAP, and an overall decrease in cellular tension, consistent with the expected outcomes of ROCK inhibition. The atypical effects of ROCKi alone may be attributable to the presence of growth-promoting factors such as insulin and hydrocortisone in the basal media, which could have partially antagonized the inhibitory effects of ROCKi. The addition of EGF or STO-CM may have reestablished the expected cellular response to ROCK inhibition. These findings suggest a complex interplay between ROCK signaling and basal media components, warranting further investigation [73,74].

Moreover, VF epithelial cells maintained under optimized conditions with EGF or STO-CM exhibited a typical cuboidal epithelial morphology, uniform cytoskeletal organization (F-actin, cytokeratin), and minimal focal adhesion formation—features associated with a low-tension, proliferative state. In contrast, withdrawal of EGF or STO-CM induced a senescent phenotype, characterized by extensive cell spreading, robust stress fibers, and increased focal adhesion formation, all indicative of heightened cytoskeletal tension [75]. These observations collectively highlight tension-dependent morphological and cytoskeletal transitions in VF epithelial cells that are strongly influenced by culture conditions. Although direct analysis of NMII activation is beyond the scope of this study, our findings—based on traction force measurements, YAP subcellular localization, P63 expression, and paxillin/pFAK focal adhesion analyses—clearly support the conclusion that VF epithelial cell fate is governed by mechanical cues that are likely mediated via NMII-dependent pathways [76].

## 5. Conclusions

Among the growth factors incorporated into our medium formulation, we explored the effects of ROCKi, EGF, and STO-CM. With ROCKi alone, rbVFEs succumbed to growth arrest. Further enhancement of EGF or STO-CM is necessary for the long-term expansion of rbVFEs. Moreover, EGF was equipotent to STO-CM in enhancing rbVFE proliferation, providing a convenient alternative to STO-CM and assisting in feeder-free systems. In the absence of EGF or STO-CM in the medium, nuclear YAP shuttling was observed, leading to senescent morphological features, such as increased actin stress fibers and mature focal adhesions, leading to increased cellular area and cellular forces. However, the incorporation of EGF and STO-CM to ROCKi-containing media significantly reversed these phenomena and promoted the proliferation and self-renewal of rbVFEs. Furthermore, by incorporating biophysical analyses TFM and IFM, we demonstrated correlation between cellular forces and biological phenomena (proliferation, self-renewal, and senescence). In summary, our newly devised medium, enriched with ROCKi, EGF, or STO-CM, can promote the long-term expansion of rbVFEs by remodeling the rbVE cytoskeleton to a low-cellular-force state, thus inhibiting cellular senescence and promoting cell proliferation. Our cell culture strategy can provide an abundant supply of epithelial cells for VF mucosal research. Decreased immunogenicity and potential immunosuppressive properties associated with VF-derived adult stem cells can facilitate allogeneic transplantation, providing an advantage as a cell source for VF regenerative medicine.

## Data Availability

The original contributions presented in this study are included in the article. Further inquiries can be directed to the corresponding authors.

## Supplementary Materials

The following supporting information can be downloaded at: https://www.mdpi.com/article/10.3390/cells14181412/s1, Figure S1: Hematoxylin and Eosin (H & E) and Masson’s Trichrome (MT) staining of rabbit VF tissue to visualize the tissue structure. Scale bar = 600 µm, 150 µm, 75 µm from left to right; Figure S2: Adhesion based purification of VF epithelial cells and fibroblasts. (**A**) Schematic to demonstrate fibroblasts removal after short-term trypsin treatment to mixture of epithelial cells and fibroblasts. (**B**) Phase contrast images of rbVFEs before and after trypsin treatment for 1–2 min. Scale bar = 300 µm. (**C**) IF staining with epithelial marker Cytokeratin 14 (CK14) to characterize rbVFEs purified with adhesion-based technique. Scale bar = 300 µm; Figure S3: STO feeder vs STO conditioned media (STO-CM) for supporting rbVFEs proliferation. (**A**) Phase contrast images and (**C**) daily absorbance value of rbVFEs cultured in STO feeder or STO-CM. Scale bar = 300 µm and 50 µm (cropped). Data are presented as the mean ± SD, * *p* < 0.05, ** *p* < 0.01, *** *p* < 0.001 using two-way ANOVA test followed by Tukey’s multiple comparisons test. (**B**) Click iT Edu images and (**D**) quantification of rbVFEs in S-phase when cultured with STO feeder or STO-CM. Scale bar = 150 µm and 50 µm (cropped). Data are presented as the mean ± SD, * *p* < 0.05, ** *p* < 0.01, *** *p* < 0.001 using one-way ANOVA test followed by Tukey’s multiple comparisons test; Figure S4: Epidermal Growth Factor (EGF) at 5 ng/mL can replace STO feeder cells for the long-term expansion of rbVFEs. (**A**) Phase-contrast images of rbVFEs cultured under three conditions: (i) 0.125 ng/mL EGF only, (ii) STO feeder with 0.125 ng/mL EGF, and (iii) 5 ng/mL EGF only. Scale bar = 300 µm. (**B**) Quantification of population doublings, cumulative population doublings, and population doubling time under each condition. All three conditions are supplemented by ROCKi. For the 1st group (0.125 ng/mL EGF only), *n* = 1 biological specimen. For the 2nd (STO feeder + 0.125 ng/mL EGF) and 3rd groups (5 ng/mL EGF only), *n* = 2 biological specimens. rbVFEs used for the experiment were purified with adhesion-based technique only; Figure S5: YAP nuclear translocation and pFAK based focal adhesion formation in rbVFEs when cultured with different combinations of growth factors (ROCKi, EGF and STO-CM) in different substrate, tissue culture plate (TCP) and 1.8 kPa PAA gel. (**A**) Matlab based intensity analysis of YAP, pFAK (both in the form of focal adhesion [FA] and dispersed throughout the cell [Cell]), and F-actin, when rbVFEs are cultured in TCP. (**B**) Matlab based intensity analysis of YAP, pFAK (both in the form of focal adhesion [FA] and dispersed throughout the cell [Cell]), and F-actin, when rbVFEs are cultured in 1.8 kPa PAA gel. Scale bar = 50 µm; Figure S6: P63 intensity calculations for rbVFEs cultured with different combinations of growth factors (ROCKi, EGF and STO-CM) in different substrate, tissue culture plate (TCP) and 1.8 kPa PAA gel. (**A**) IF staining and respective matlab based intensity analysis of DAPI and P63 for rbVFEs cultured in TCP. (**B**) IF staining and respective matlab based intensity analysis of DAPI and P63 for rbVFEs cultured in 1.8 kPa PAA gel. Scale bar = 150 µm; Figure S7: Paxillin based focal adhesion formation in rbVFEs when cultured with different combinations of growth factors (ROCKi, EGF and STO-CM) in different substrate, tissue culture plate (TCP) and 1.8 kPa PAA gel. (**A**) Matlab based intensity analysis of paxillin (both in the form of focal adhesion [FA] and dispersed throughout the cell [Cell]), and F-actin, when rbVFEs are cultured in TCP. (**B**) Matlab based intensity analysis of paxillin (both in the form of focal adhesion [FA] and dispersed throughout the cell [Cell]), and F-actin, when rbVFEs are cultured in 1.8 kPa PAA gel. Scale bar = 50 µm; Figure S8: Anti-apoptosis marker expression and cytoskeletal remodeling in rbVFEs when cultured with different combinations of growth factors (ROCKi, EGF and STO-CM) in different substrate, tissue culture plate (TCP) and 1.8 kPa PAA gel. (**A**) Matlab based intensity analysis of BCL-2, CK-14 and F-actin, when rbVFEs are cultured in TCP; (**B**) Matlab based intensity analysis of BCL-2, CK-14 and F-actin, when rbVFEs are cultured 1.8 kPa PAA gel. Scale bar = 50 µm; Figure S9: YAP nuclear/cytoplasmic localization under different biochemical and mechanical cues. (**A**) Representative immunofluorescence images of YAP (red), nuclei (Hoechst, blue), and merged channels in cells cultured on standard TCP with different combinations of growth factors (ROCKi, EGF and STO-CM). (**B**) Cells cultured on soft polyacrylamide (PAA) gels (1.8 kPa), mimicking a more physiologically relevant soft substrate. The schematic above each condition illustrates expected YAP localization driven by cytoskeletal tension and focal adhesion formation). Data are plotted as individual points, with the line representing the mean value, * *p* < 0.05, ** *p* < 0.01, *** *p* < 0.001 using one-way ANOVA test followed by Tukey’s multiple comparisons test. Abbreviations: TCP, tissue culture plastic. Scale bar = 25 µm; Table S1: List of primers used for qPCR; Table S2: List of primary antibodies; Table S3: List of secondary antibodies.

## Abbreviations

The following abbreviations are used in this manuscript:

VF	Vocal fold
VFEs	VF-derived epithelial cells
rb	Rabbit
ROCKi	Rho kinase inhibitor
EGF	Epidermal growth factor
STO	SIM mouse embryo-derived thioguanine and ouabain-resistant (STO) fibroblasts
CM	Conditioned media
YAP	Yes-associated protein
FA	Focal adhesion
FAK	Focal adhesion kinase
Krt	Keratin
iPSCs	Induced pluripotent stem cells
ECM	Extracellular matrix
PD	Population doubling
PAA	Polyacrylamide
TFM	Traction force microscopy
IFM	Intracellular force microscopy
TGF-β	Transforming growth factor β
bFGF	Basic fibroblast growth factor
IGF-1	Insulin-like growth factor 1
HGF	Hepatocyte growth factor
VEGF	Vascular endothelial growth factor
IGFBP-6	Insulin-like growth factor binding protein
CSF-1	Macrophage colony-stimulating factor
PEDF	Pigment epithelium-derived factor
SCF	Stem cell factor
IFN-γ	Interferon gamma
NMII	Non-muscle myosin II
ROCK	Rho kinase
