# Supplementary material for: Mechanotransduction-Mediated Expansion of Rabbit Vocal Fold Epithelial Cells via ROCK Inhibition and Stromal Cell-Derived Paracrine Signals
Source: Cells. 2025 Sep 9;14(18):1412. doi: 10.3390/cells14181412 (PMC12468236; doi:10.3390/cells14181412)
Supplement: Supplementary file 1 [file cells-14-01412-s001.zip › cells-3804644-supplementary.pdf]

Supporting Information

Mechanotransduction-mediated Expansion of Rabbit Vocal Fold Epithelial Cells via ROCK Inhibition and Stromal Cell-Derived Paracrine Signals

Samjhana Thapa[1,2,†], Joo Hyun Kim[1,3,†], Jun Yeong Jeong[4], Sung Sik Hur[1], Seung Won Lee[4,*], and Yongsung Hwang[1,2,*]

[1]Soonchunhyang Institute of Medi-bio Science (SIMS), Soonchunhyang University, Cheonan-si, Chungcheongnam-do 31151, Republic of Korea; samjhanathp@gmail.com (S.T.); noirsky@naver.com (J.H.K.); sstahur@gmail.com (S.S.H.)
[2]Department of Integrated Biomedical Science, Soonchunhyang University, Asan-si, Chungcheongnam-do 31538, Republic of Korea
[3]Department of Otorhinolaryngology-Head and Neck Surgery, College of Medicine, Soonchunhyang University, Cheonan Hospital, Republic of Korea
[4]Department of Otorhinolaryngology-Head and Neck Surgery, College of Medicine, Soonchunhyang University, Bucheon Hospital, Republic of Korea; 136076@schmc.ac.kr (J.Y.J.)

* Correspondence: lsw0922@schmc.ac.kr (S.W.L.); yshwang0428@sch.ac.kr (Y.H.)
† These authors contributed equally to this work.

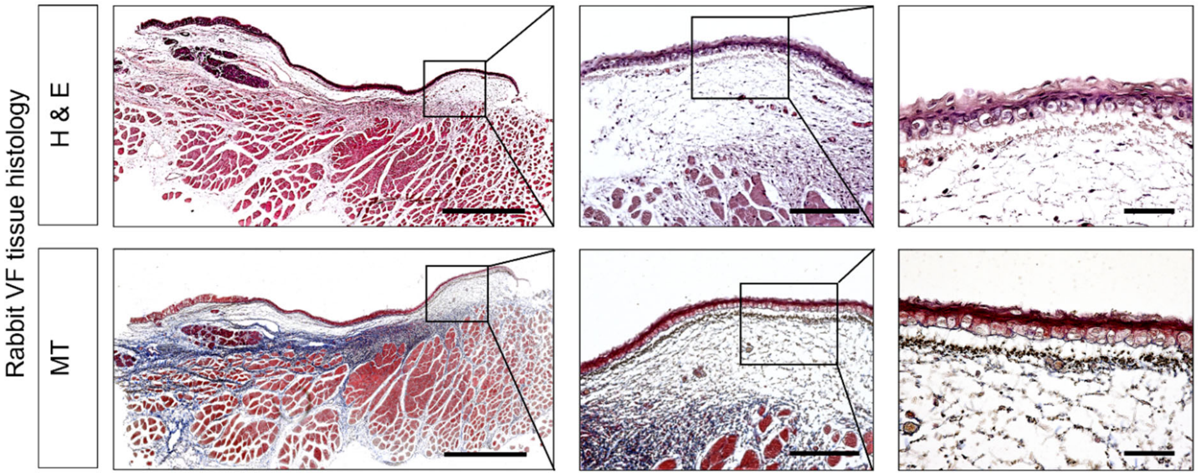

Figure S1. Hematoxylin and Eosin (H & E) and Masson's Trichrome (MT) staining of rabbit VF tissue to visualize the tissue structure. Scale bar = 600 μm, 150 μm, 75 μm from left to right.

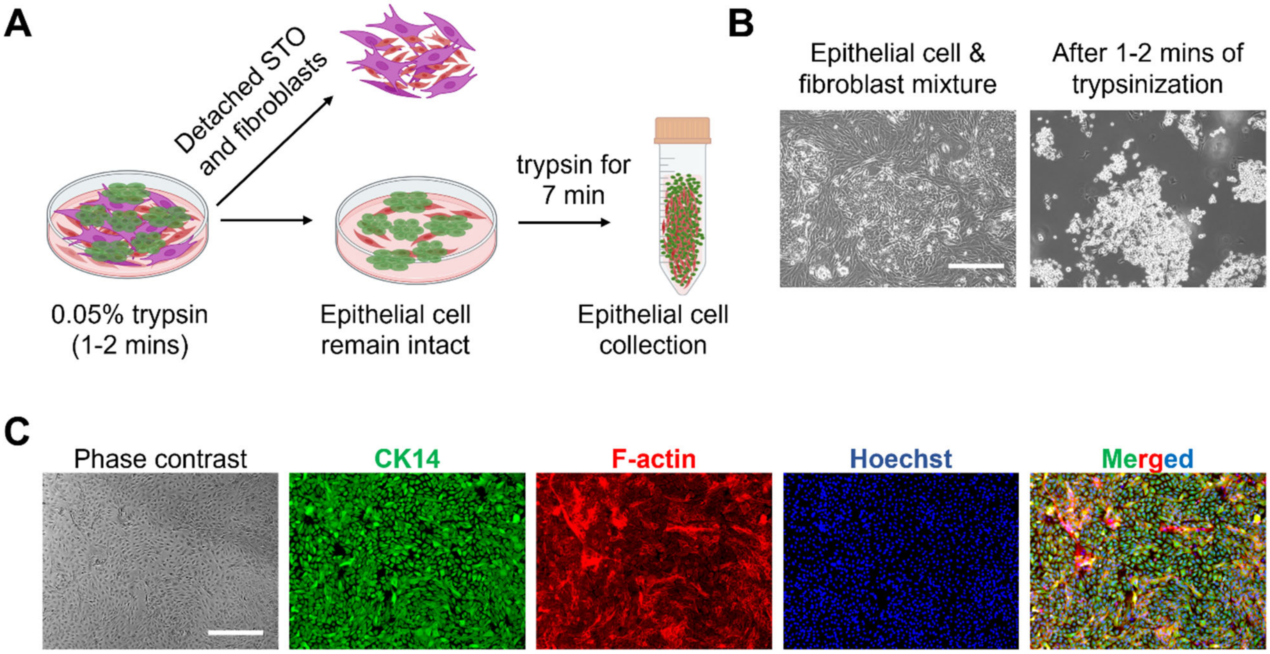

Figure S2. Adhesion based purification of VF epithelial cells and fibroblasts. (**A**) Schematic to demonstrate fibroblasts removal after short-term trypsin treatment to mixture of epithelial cells and fibroblasts. (**B**) Phase contrast images of rbVFEs before and after trypsin treatment for 1–2 min. Scale bar = 300 μm. (**C**) IF staining with epithelial marker Cytokeratin 14 (CK14) to characterize rbVFEs purified with adhesion-based technique. Scale bar = 300 μm.

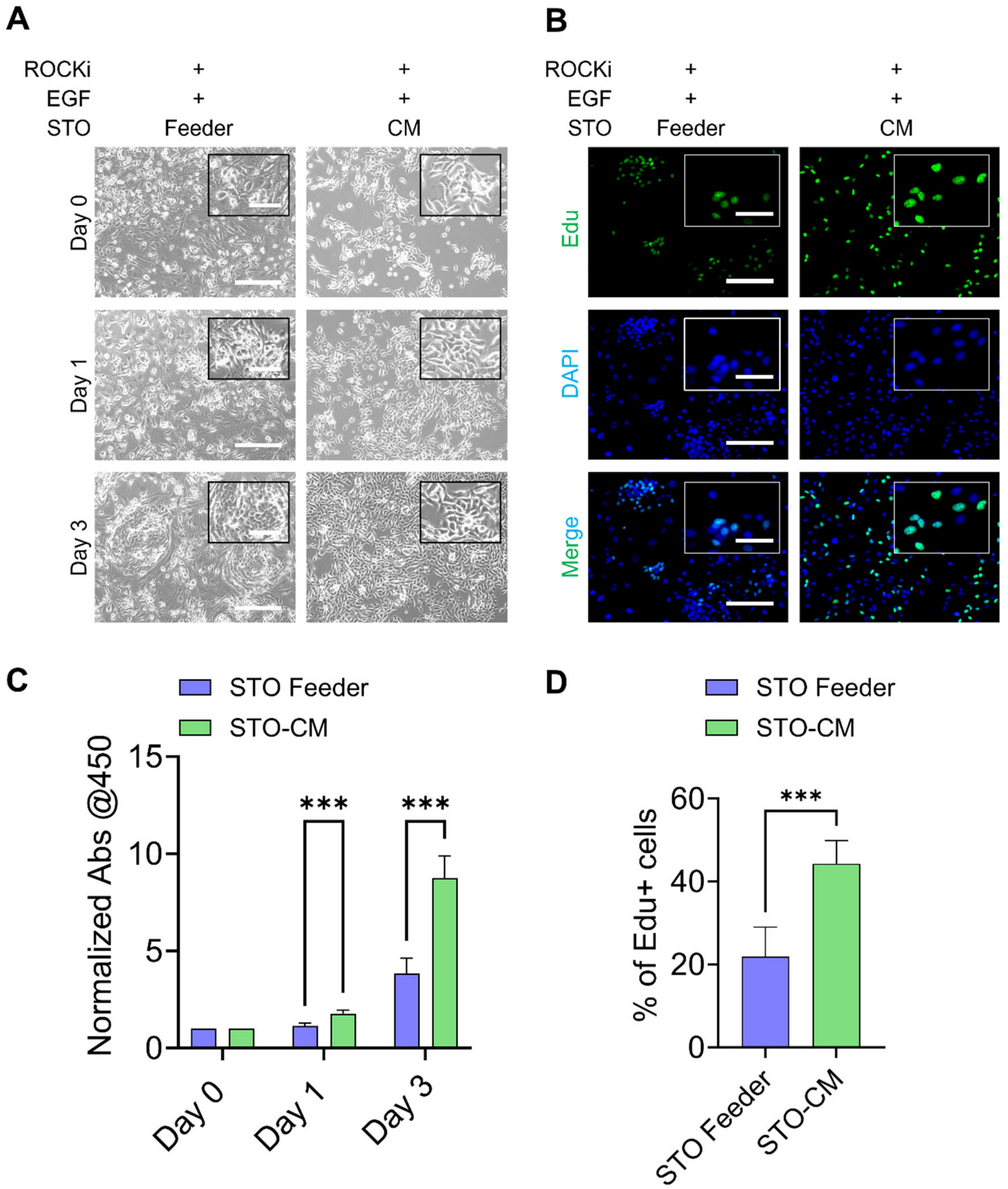

Figure S3. STO feeder vs STO conditioned media (STO-CM) for supporting rbVFEs proliferation. (**A**) Phase contrast images and (**C**) daily absorbance value of rbVFEs cultured in STO feeder or STO-CM. Scale bar = 300 μm and 50 μm (cropped). Data are presented as the mean ± SD, * $p < 0.05$, ** $p < 0.01$, *** $p < 0.001$ using two-way ANOVA test followed by Tukey's multiple comparisons test. (**B**) Click iT Edu images and (**D**) quantification of rbVFEs in S-phase when cultured with STO feeder or STO-CM. Scale bar = 150 μm and 50 μm (cropped). Data are presented as the mean ± SD, * $p < 0.05$, ** *p* <

0.01, *** $p < 0.001$ using one-way ANOVA test followed by Tukey's multiple comparisons test.

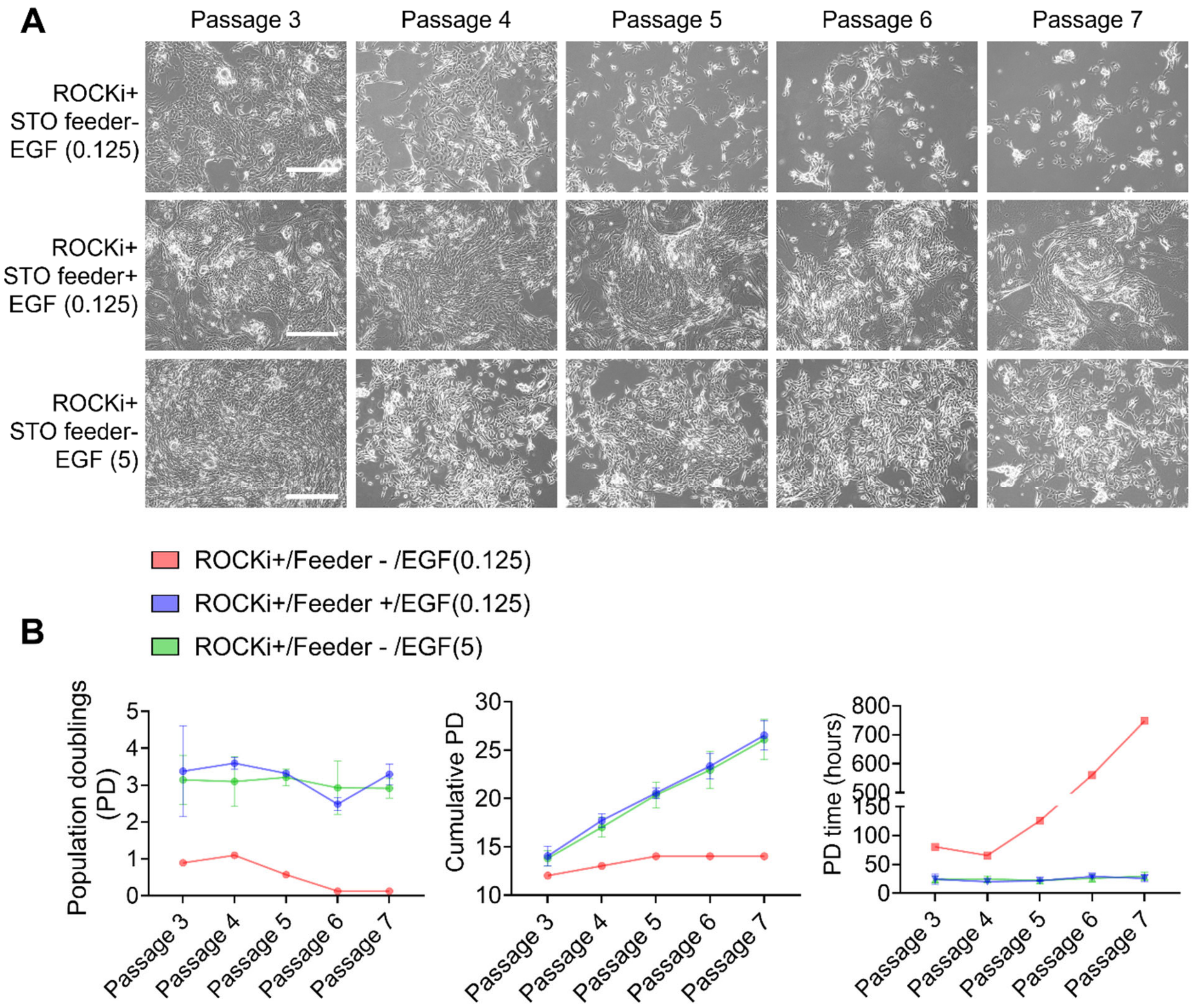

Figure S4. Epidermal Growth Factor (EGF) at 5 ng/mL can replace STO feeder cells for the long-term expansion of rbVFEs. (**A**) Phase-contrast images of rbVFEs cultured under three conditions: (i) 0.125 ng/mL EGF only, (ii) STO feeder with 0.125 ng/mL EGF, and (iii) 5 ng/mL EGF only. Scale bar = 300 µm. (**B**) Quantification of population doublings, cumulative population doublings, and population doubling time under each condition. All three conditions are supplemented by ROCKi. For the 1st group (0.125 ng/mL EGF only), *n* = 1 biological specimen. For the 2nd (STO feeder + 0.125 ng/mL EGF) and 3rd groups (5 ng/mL EGF only), *n* = 2 biological specimens. rbVFEs used for the experiment were purified with adhesion-based technique only.

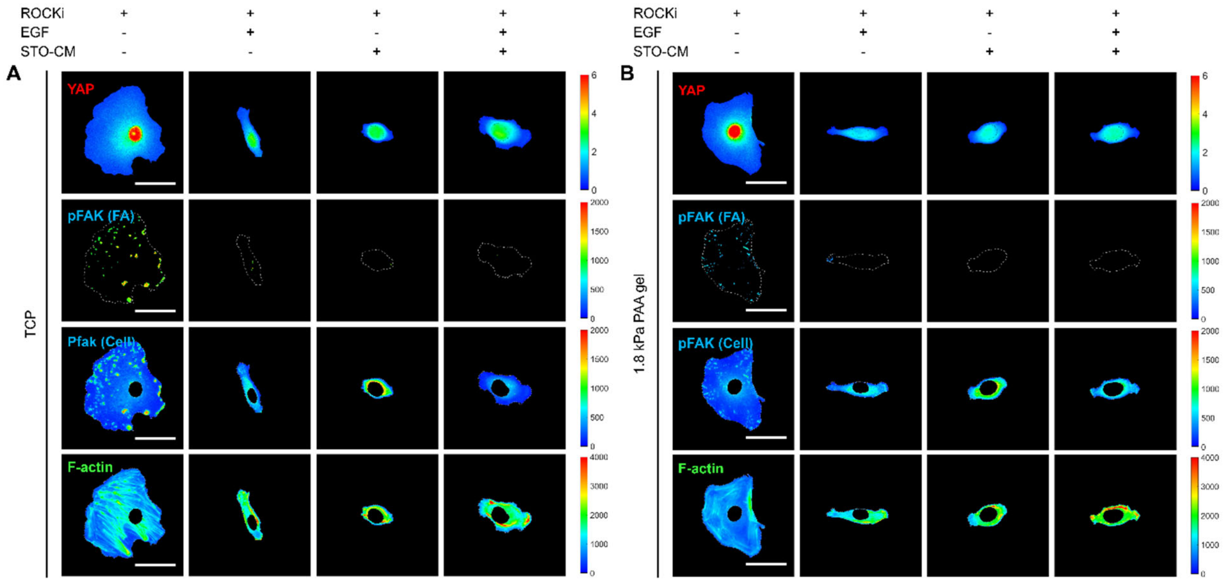

Figure S5. YAP nuclear translocation and pFAK based focal adhesion formation in rbVFEs when cultured with different combinations of growth factors (ROCKi, EGF and STO-CM) in different substrate, tissue culture plate (TCP) and 1.8 kPa PAA gel. (**A**) Matlab based intensity analysis of YAP, pFAK (both in the form of focal adhesion [FA] and dispersed throughout the cell [Cell]), and F-actin, when rbVFEs are cultured in TCP. (**B**) Matlab based intensity analysis of YAP, pFAK (both in the form of focal adhesion [FA] and dispersed throughout the cell [Cell]), and F-actin, when rbVFEs are cultured in 1.8 kPa PAA gel. Scale bar = 50 µm.

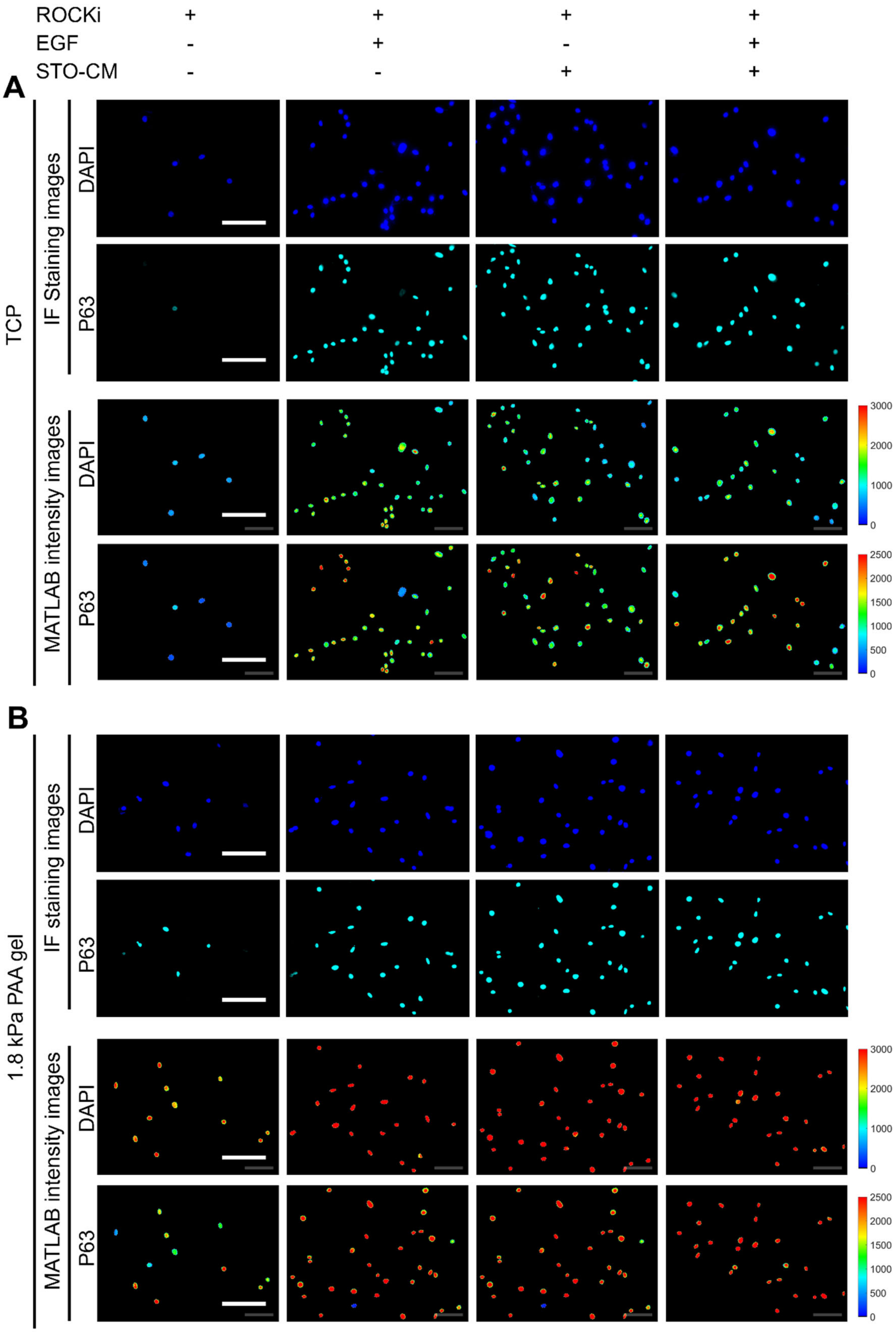

ROCKi + + + +
EGF - + - +
STO-CM - - + +
A
TCP
IF Staining images
DAPI
P63
MATLAB intensity images
DAPI
P63
3000
2000
1000
0
2500
2000
1500
1000
500
0
B
1.8 kPa PAA gel
IF staining images
DAPI
P63
MATLAB intensity images
DAPI
P63
3000
2000
1000
0
2500
2000
1500
1000
500
0

Figure S6. P63 intensity calculations for rbVFEs cultured with different combinations of growth factors (ROCKi, EGF and STO-CM) in different substrate, tissue culture plate (TCP) and 1.8 kPa PAA gel. (**A**) IF staining and respective matlab based intensity analysis of DAPI and P63 for rbVFEs cultured in TCP. (**B**) IF staining and respective matlab based intensity analysis of DAPI and P63 for rbVFEs cultured in 1.8 kPa PAA gel. Scale bar = 150 μm.

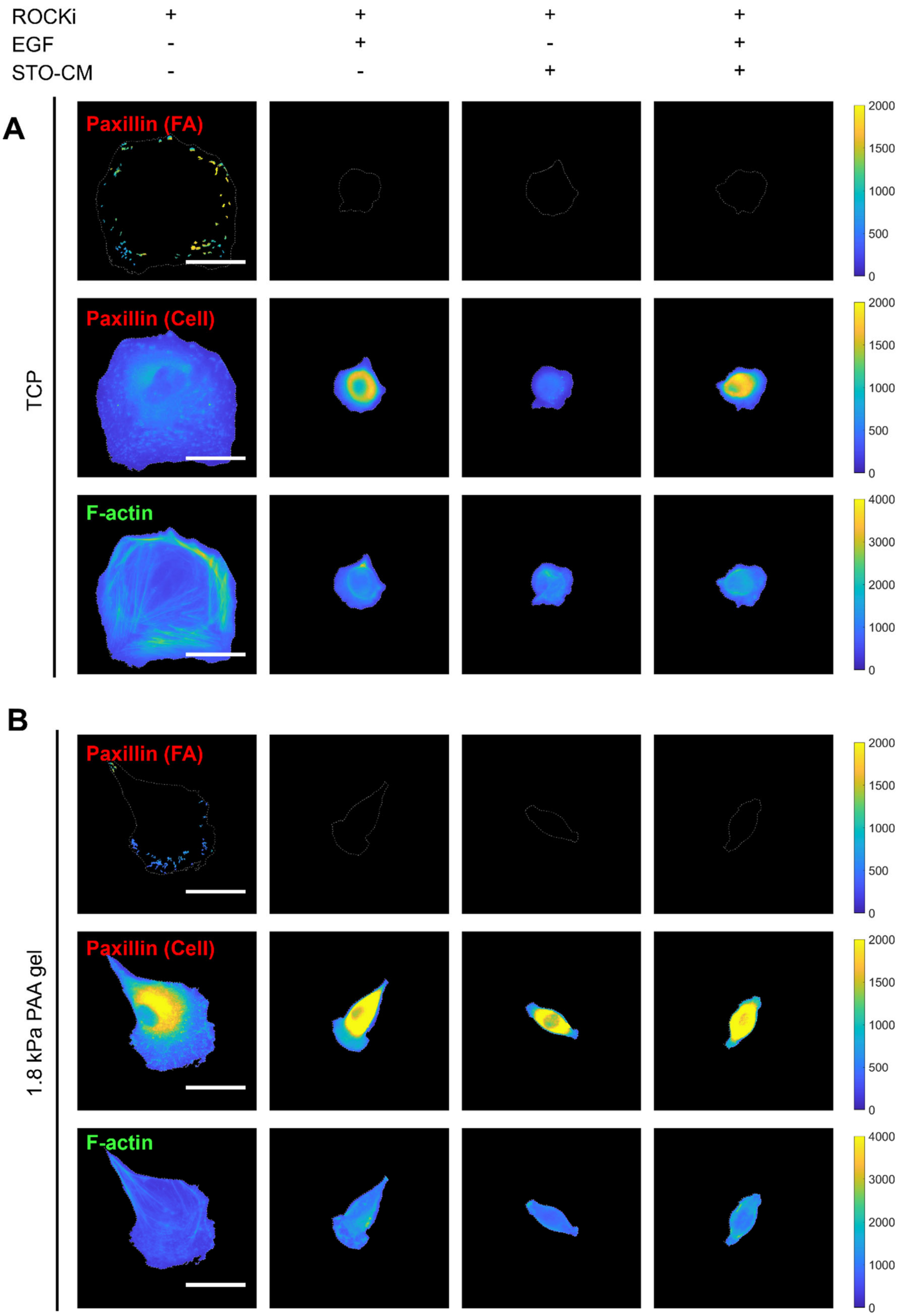
ROCKi + + + +
EGF - + - +
STO-CM - - + +
A
B
TCP
1.8 kPa PAA gel
Paxillin (FA)
Paxillin (Cell)
F-actin
2000
1500
1000
500
0
4000
3000
2000
1000
0

Figure S7. Paxillin based focal adhesion formation in rbVFEs when cultured with different combinations of growth factors (ROCKi, EGF and STO-CM) in different substrate, tissue culture plate (TCP) and 1.8 kPa PAA gel. (**A**) Matlab based intensity analysis of paxillin (both in the form of focal adhesion [FA] and dispersed throughout the cell [Cell]), and F-actin, when rbVFEs are cultured in TCP. (**B**) Matlab based intensity analysis of paxillin (both in the form of focal adhesion [FA] and dispersed throughout the cell [Cell]), and F-actin, when rbVFEs are cultured in 1.8 kPa PAA gel. Scale bar = 50 μm.

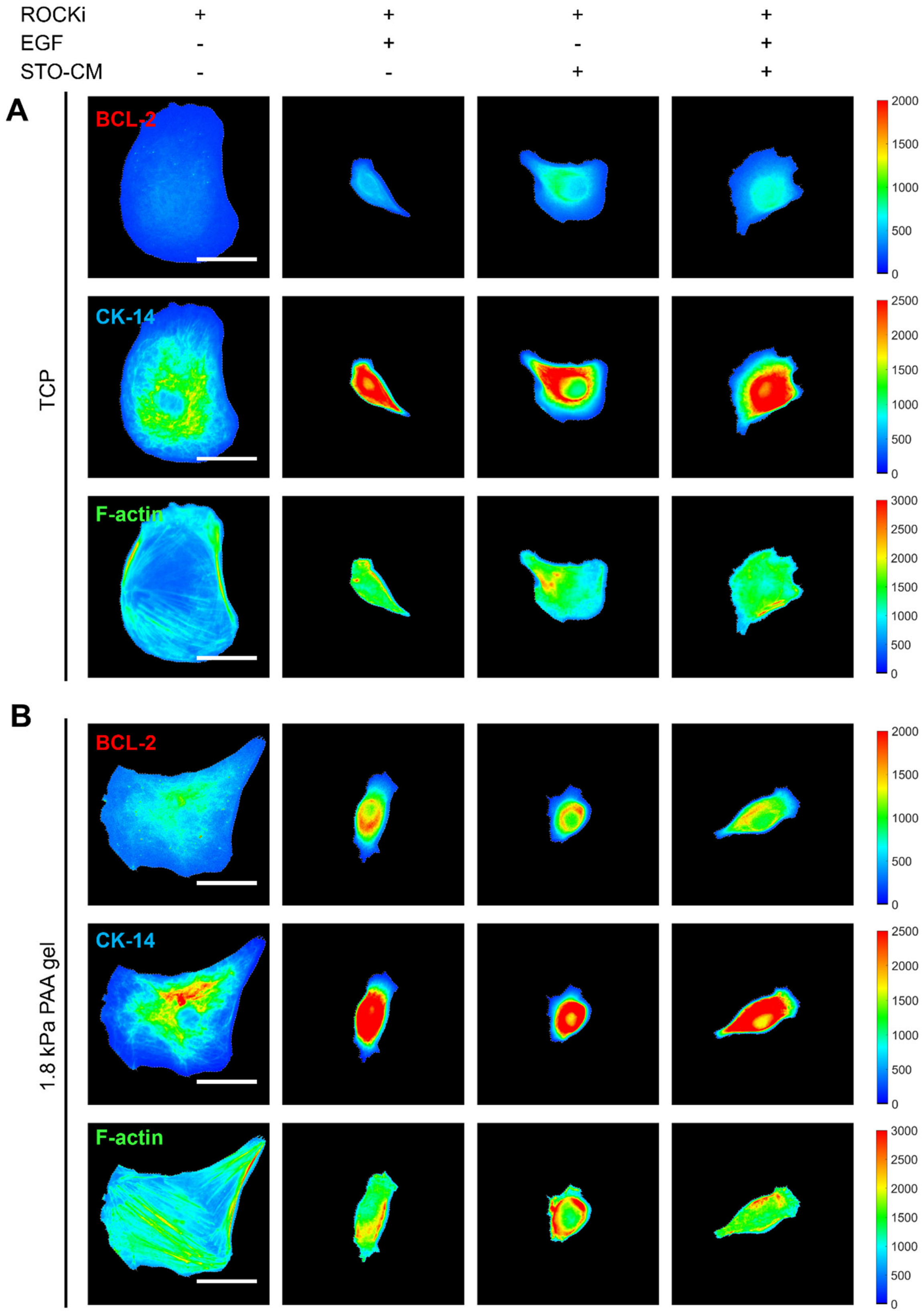
ROCKi + + + +
EGF - + - +
STO-CM - - + +
A
B
TCP
1.8 kPa PAA gel
BCL-2
CK-14
F-actin
0
500
1000
1500
2000
2500
3000

Figure S8. Anti-apoptosis marker expression and cytoskeletal remodeling in rbVFEs when cultured with different combinations of growth factors (ROCKi, EGF and STO-CM) in different substrate, tissue culture plate (TCP) and 1.8 kPa PAA gel. (**A**) Matlab based intensity analysis of BCL-2, CK-14 and F-actin, when rbVFEs are cultured in TCP; (**B**) Matlab based intensity analysis of BCL-2, CK-14 and F-actin, when rbVFEs are cultured 1.8 kPa PAA gel. Scale bar = 50 μm.

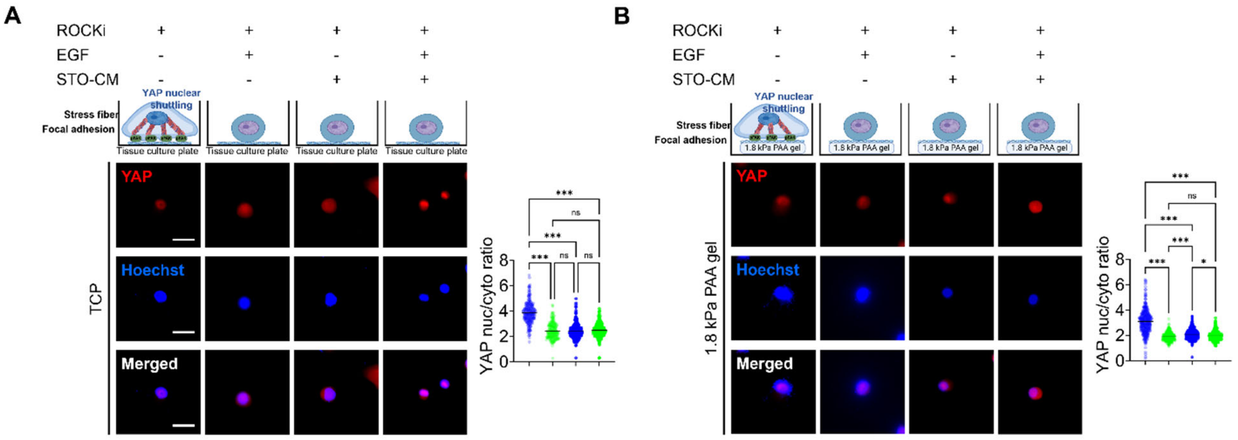

Figure S9. YAP nuclear/cytoplasmic localization under different biochemical and mechanical cues. (**A**) Representative immunofluorescence images of YAP (red), nuclei (Hoechst, blue), and merged channels in cells cultured on standard TCP with different combinations of growth factors (ROCKi, EGF and STO-CM). (**B**) Cells cultured on soft polyacrylamide (PAA) gels (1.8 kPa), mimicking a more physiologically relevant soft substrate. The schematic above each condition illustrates expected YAP localization driven by cytoskeletal tension and focal adhesion formation). Data are plotted as individual points, with the line representing the mean value, * $p < 0.05$, ** $p < 0.01$, *** $p < 0.001$ using one-way ANOVA test followed by Tukey's multiple comparisons test. Abbreviations: TCP, tissue culture plastic. Scale bar = 25 μm.

Supplementary Table S1. List of primers used for qPCR.

Gene Name	Primer Sequence (5' to 3')
**GAPDH**	F - AGACACGATGGTGAAGGTCG
entry 2	R - TGCCGTGGGTGGAATCATAC
**P63**	F - CAGAAAGCAGCAAGTGTCGG
	R - ATGGGTGTTCTGACGAAAGGG
**PCNA**	F - GCACGTATATGCCGAGACCT
	R - CACTGGCGGAAAACTTCACC
**CDKN2A**	F -CTCCTCAGAGACACGTGAGCG
	R - TGACCTTACGCTCGCTCCT
**P53**	F - TGCTGATCATGCCTACCTCAC
	R - GTAGGGAACCAGCACCATGAG
**β-galactosidase**	F - AGATCGACTACAGCCGGGAC
	R - GAAGTAGTGGATGCTCCCCG
**KRT 14**	F - GTTCTCCTCGGGCTCTCAGTC
	R - GGTGCGGGGCAGTCTTAGTT

**KRT 17**	F - CACCAGACCACCCGCTAAG
	R - AGGGACTGAAGCGAGGACTG
**YAP**	F - CCCTCGTTTTGGTAAAGCCAT
	R - GTTGCTGCTGGTTGGAACTG
**CCN1**	F - AGACTTGTGGAACCGGCATC
	R - CATTTCTTGCCCTTTTTCAGGC
**CCN2**	F - GCGTGTGCACCGCCAAAGAT
	R - CGCCATCCAGGCAAGTGCA
**WWTR1**	F - CCTGGGGTTAGGTTGCTACA
	R - GCCCTGCATTTTCTCCTGTGT

Supplementary Table S2. List of primary antibodies.

Primary Antibody	Company	Dilution	Catalog No.	Host Species	Application
CD 44	Thermofisher Scientific	1:50	MA4400	Rat	MACS labelling
MTS-1	Santa Cruz Biotechnology	1:100	sc377059	Mouse	IF
CK-14	Abcam	1:200	ab119695	Rabbit	IF
Paxillin	Thermofisher Scientific	5:200	MA5-13356	Mouse	IF
P63	Abcam	1:200	ab124762	Rabbit	IF
Bcl-2	Santa Cruz Biotechnology	1:100	sc-7382	Mouse	IF
YAP	Santa Cruz Biotechnology	1: 200	sc-101199	Mouse	IF
pFAK	Abcam	1:400	ab81298	Rabbit	IF

Supplementary Table S3. List of secondary antibodies.

Secondary Antibody	Company	Dilution	Catalog No.	Fluorophore /Label	Host Species	Application
Goat anti-rabbit	Thermofisher Scientific	1:200	A11008	Alexa Fluor 488	Goat	IF
Goat anti-mouse	Thermofisher Scientific	1:200	A21422	Alexa Fluor 555	Goat	IF
Donkey anti-rabbit	Thermofisher Scientific	1:200	A31573	Alexa Fluor 647	Donkey	IF
Phalloidin	Thermofisher Scientific	1:400	A22287	Alexa Fluor 647	-	IF
Phalloidin	Thermofisher Scientific	1:400	A12379	Alexa Fluor 488	-	IF
Hoechst	Thermofisher Scientific	1:2000	H21492	-	-	IF
